# MYC Oncogene: A Druggable Target for Treating Cancers with Natural Products

**DOI:** 10.14336/AD.2023.0520

**Published:** 2024-04-01

**Authors:** Ka Iong Chan, Siyuan Zhang, Guodong Li, Yida Xu, Liao Cui, Yitao Wang, Huanxing Su, Wen Tan, Zhangfeng Zhong

**Affiliations:** ^1^Macao Centre for Research and Development in Chinese Medicine, State Key Laboratory of Quality Research in Chinese Medicine, Institute of Chinese Medical Sciences, University of Macau, Macao SAR 999078, China; ^2^Guangdong Provincial Key Laboratory of Research and Development of Natural Drugs, School of Pharmacy, Guangdong Medical University, Zhanjiang 524000, China; ^3^School of Pharmacy, Lanzhou University, Lanzhou 730000, China

**Keywords:** MYC, cancer, immune response, multidrug resistance, natural product, herbal medicine

## Abstract

Various diseases, including cancers, age-associated disorders, and acute liver failure, have been linked to the oncogene, *MYC*. Animal testing and clinical trials have shown that sustained tumor volume reduction can be achieved when MYC is inactivated, and different combinations of therapeutic agents including MYC inhibitors are currently being developed. In this review, we first provide a summary of the multiple biological functions of the MYC oncoprotein in cancer treatment, highlighting that the equilibrium points of the MYC/MAX, MIZ1/MYC/MAX, and MAD (MNT)/MAX complexes have further potential in cancer treatment that could be used to restrain MYC oncogene expression and its functions in tumorigenesis. We also discuss the multifunctional capacity of MYC in various cellular cancer processes, including its influences on immune response, metabolism, cell cycle, apoptosis, autophagy, pyroptosis, metastasis, angiogenesis, multidrug resistance, and intestinal flora. Moreover, we summarize the MYC therapy patent landscape and emphasize the potential of MYC as a druggable target, using herbal medicine modulators. Finally, we describe pending challenges and future perspectives in biomedical research, involving the development of therapeutic approaches to modulate MYC or its targeted genes. Patients with cancers driven by MYC signaling may benefit from therapies targeting these pathways, which could delay cancerous growth and recover antitumor immune responses.

## 1. Introduction

**Figure 1.**
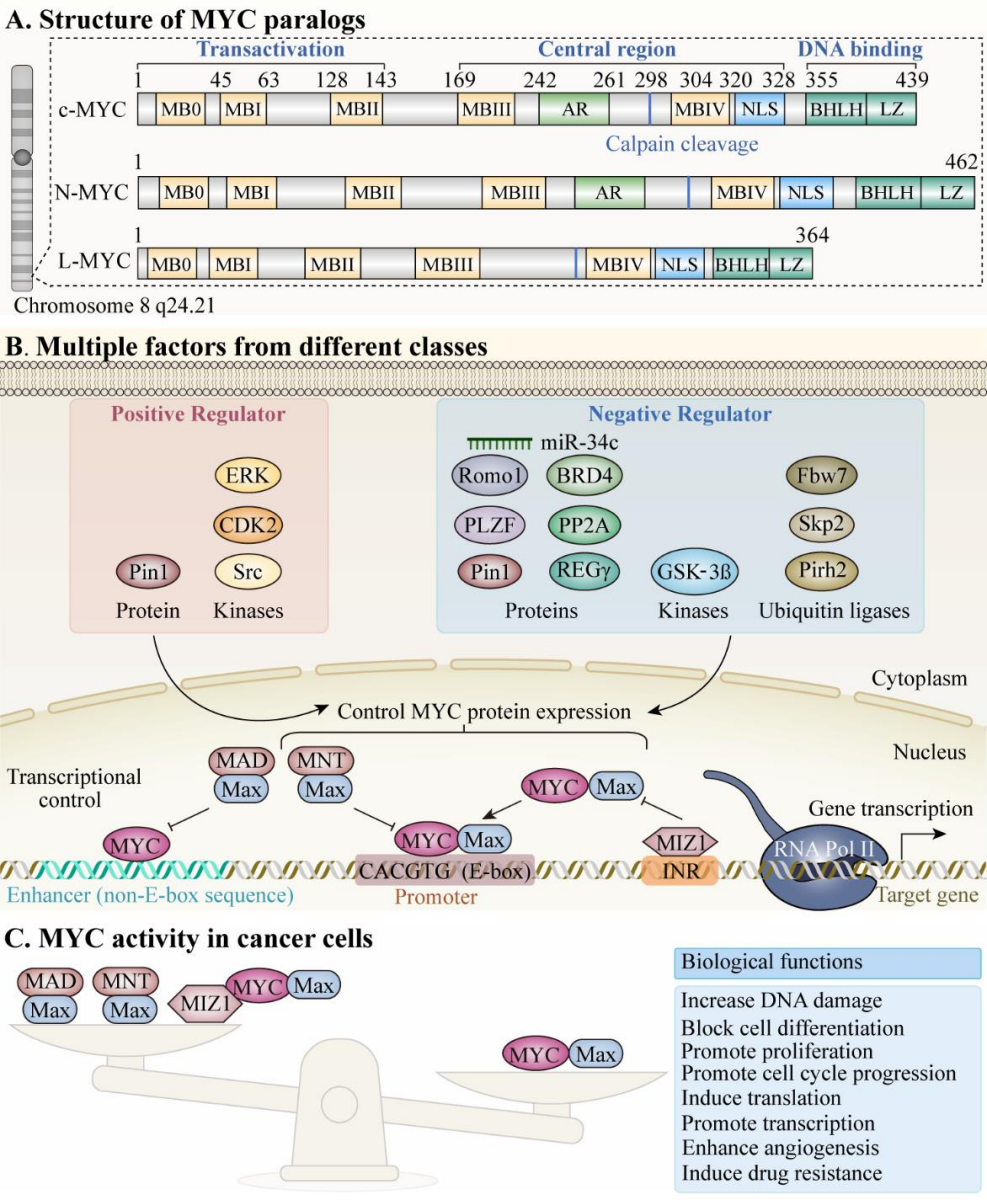
**Schematic representations of the functional regions of three MYC paralogs**. The N terminus of MYC comprises a transactivation domain locates on residues 1-143 and three highly conserved elements: MYC boxes (MB) 1-3 (MBI: residues 45-63; MBII: 128-143; MBIII: 169-199). FBW7 targets the phosphodegron in MBI. MBII recruits a histone acetyltransferase (HAT) complex that mediates all known MYC functions. MBIII is key to regulating the stability and transcription level of MYC. The BR/HLH/LZ motif (residues 355-439) at the C-terminus is necessary for DNA binding and binding to its canonical partner, MAX. (B) Summary of different classes of positive and negative regulatory factors that influence regulation of MYC expression networks. The transcriptional activities of target genes are driven by the MYC/MAX dimer, which bind to DNA E-boxes or non-E-box sequences. (C) Interactions among MYC, MIZ1, MAX, and MAD (or MNT), different combinations of which exhibit varying effects on transcriptional activity. Disruption of the balance among these complexes can influence significant biological functions and exert cancerous effects in tumorigenesis. BRD4: Bromodomain-containing protein 4; CDK2: Cyclin-dependent kinase 2; ERK: Extracellular signal-regulated kinase; Fbw7: F-box and WD repeat domain containing 7; GSK-3β: Glycogen Synthase Kinase 3 Beta; MAX: MYC Associated Factor X; MIZ1: Myc-interacting zinc finger protein 1; MNT: MAX Network Transcriptional Repressor; Pin1: Peptidylprolyl Cis/Trans Isomerase, NIMA-Interacting 1; Pirh2: p53-induced RING-H2 protein; PLZF: promyelocytic leukemia zinc finger; PP2A: Protein Phosphatase 2A; REGγ: REGgamma proteasome; Romo1: Reactive Oxygen Species (ROS) Modulator 1; Skp2: S-Phase Kinase Associated Protein 2.

MYC is a “global” transcription factor that contributes to various diseases, including cancers, age-associated disorders, and acute liver failure, among others. Due to its involvement in multiple cellular processes, including DNA repair, protein translation, cell cycle arrest, stress response, cellular proliferation and differentiation, programmed cell death, immune response regulation and stem cell differentiation, MYC is referred to as a “master gene regulator”; it is thought to regulate approximately 15% of the human genome [1-4], and functions by controlling RNA polymerases to regulate transcription levels [5-7]. Among basic helix-loop-helix leucine zipper DNA binding proteins, c-MYC, N-MYC, and L-MYC comprise the MYC transcription factor (TF) subfamily, which is encoded on chromosome 8q24.21 (Fig. 1A) [8-10].

MYC is among the most widely investigated cancer-causing genes, and is implicated in the formation, maintenance, and progression of various cancer types; approximately 70% of human cancers are associated with dysregulated MYC expression [11, 12]. The oncogenic effects of MYC appear to depend on cellular context and contribute to downstream pathways, including oxidative stress, the Warburg effect, and the immune microenvironment, as well as ubiquitin ligases, immune checkpoints, and ATP-binding cassette (ABC) transporters [10, 13]. Besides DNA repair and protein translation, MYC also functions in cell proliferation and survival [14, 15]. Further, MYC contributes to immune response regulation, and is associated with immune checkpoints, inducing immune evasion of MYC-mediated cancer cells and promoting tumor development [16]. Given these multiple functions of MYC in stimulating development of cancerous or precancerous cells, novel treatments targeting MYC have potential for application in patients with MYC-mediated-malignancies [8-10].

Herbal medicines have been used for thousands of years and their applications in cancer treatments have inspired interest in biochemical analysis of their functions [17-22]. Several agents have been reported that can directly or indirectly interfere with MYC expression and exhibit anticancer activity, causing tumor regression in preclinical stage studies. The objectives of this comprehensive review were to provide an overview of the roles of MYC in precancerous and cancerous cells and to discuss the design and properties of MYC inhibitors, especially modulators from medicinal herbs, that have been brought to market or are undergoing pre-clinical cancer therapy studies.

## 2. MYC Structure and Functions

### 2.1 MYC Family Protein Functional Domains

The three paralogous MYC oncoproteins, c-MYC, L-MYC, and N-MYC, have a multi-domain structure (Fig. 1A) [8-10]. All contain three distinct domains: an N-terminal trans-activating domain (TAD); a core domain responsible for maintaining nuclear stability and assisting in localization; and a C-terminal DNA binding domain that requires MYC to interact with its partner, MAX, to form MYC/MAX complexes. MYC/MAX dimerization allows integration of the E-box DNA response element, which contain a CACGTG consensus sequence, in the DNA binding domain of the complex, which can then activate and regulate gene transcription [23]. M boxes (MB) are highly conserved regions present in the MYC oncoprotein family [24, 25]. L-MYC contains five MB, where the TAD region contains MB0, MBI, and MBII, while MBIIIb and MBIV are located in the core domain [26]. C- and N-MYC include six MBs, similar to L-MYC, but with one extra, MBIV, located at their N-termini [26]. Various MYC functions are dictated by MB domain types and their interactions with corresponding proteins. MB0 promotes oncogenic transcription by interacting with transcription factor II-F (TFIIF), which contributes to transcriptional elongation [26, 27]. The MBI domain is a MYC ubiquitination site, mediating its degradation by proteasomal enzymes [25]. MBII is essential for MYC-mediated transcription activation by promoting interaction of the MYC-TRRAP-HAT complex [26]. Additionally, MBII and MB0 are critical in tumor initiation, which induce tumorigenesis, aggravating the oncogenic effects of MYC [28]. MBIIIa is associated with apoptosis [29], whereas MBIIIb interacts with the WD40-repeat protein, WDR5, to facilitate MYC-chromatin binding [30]. Interaction of MBIV with chromatin and host cell factor-1 (HCF-1) result in apoptosis and cell cycle arrest [31, 32].

### 2.2 MYC Co-factors

#### 2.2.1 MYC Positive and Negative Regulators

Various proteins interact with different MYC domains to trigger distinct functional roles (Fig. 1B).

##### 2.2.1.1 Cyclin-dependent kinase 2 (CDK2) and Extracellular receptor kinase (ERK)

CDK2 and ERK phosphorylate MYC Ser-62 to stabilize the protein, whereas glycogen synthase kinase (GSK-3β) phosphorylates Thr-58, leading to its proteasomal degradation [33].

##### 2.2.1.2 Ras-like protein (Ras)

Ras is a small GTP-binding protein, upstream of several signaling pathways including Raf/MEK/ERK and PI3K/AKT, among others. MYC production and stability can be increased by mitogenic stimulation, as can Ras activity [34]. Ras enhances MYC protein stability by phosphorylating Ser-62 via ERK [35-37]. By activating PI3K/AKT, Ras suppresses GSK-3β while stabilizing and elevating MYC levels, preventing Thr-58 phosphorylation [35-37]. Decreased Ras activity downregulates AKT signaling in late G1 phase, causing MYC degradation [38]. Ser-62 and Thr-58 are both important in regulation of cell proliferation via control of MYC expression [35-37]. Ras/Raf signaling induces MYC expression through promoter regulation [39]. Ras stimulates the Raf/MAPK/MEK signaling pathway, which allows TF activation and promotes MYC expression [40]. Mitogen-activated protein kinase (MAPK) levels can be elevated by increasing *Ras* proto-oncogene activity, which is triggered by Src kinases and platelet-derived growth factor receptors (PDGFR) [40]. The Src-PDGF axis can independently activate MYC transcription without inducing Ras [41]. Further, there is evidence that Rho proteins, including rhodopsin (Rho), Rac family small GTPase 1 (Rac), and cell division cycle 42 (Cdc42), can be upregulated by Src phosphorylation of Vav2, thereby stimulating the *MYC* promoter, and increasing MYC transcription [42].

##### 2.2.1.3 Bromodomain Protein 4 (BRD4)

BRD4 is an epigenetic reader protein of the bromodomain and extra-terminal domain (BET) family, with kinase and histone acetyltransferase (HAT) domains at its N and C-termini, respectively [43]. Similar to GSK-3β, BRD4 induces MYC destabilization by phosphorylating Thr-58 [35-37]. In contrast to GSK-3β, which is found in the cytoplasm and induces extrinsic signaling, BRD 4 is commonly found in the nucleus, and mediates homestasis of MYC levels [35-37]. To maintain stable MYC levels, a tricomplex of BRD4, ERK1, and MYC functions as a regulator, whereby MYC inhibits BRD4 HAT activity, and its kinase activity is inhibited by the ERK pathway [44].

##### 2.2.1.4 Phosphatase 2A (PP2A)

PP2A phosphatase activity regulates MYC protein levels [45]. PP2A comprises three subunits: a scaffolding subunit, a catalytic component, and a regulatory region [46], and each subunit has various isoforms, which combine to generate distinct PP2A isoforms with different regulatory effects [47]. A unique subunit of the B regulatory family, B56α, negatively regulates MYC protein function and stability [48]. PP2A complexes dephosphorylate MYC Ser-62 and regulate its turnover through proteasome-mediated degradation [49]. Additionally, the B56α subunit of PP2A can dephosphorylate GSK-3β and down-regulate MYC expression [50].

##### 2.2.1.5 Prolyl Isomerase (Pin1)

Pin1 recognizes specific phosphorylated residues (pThr-58 and pSer-62) and isomerizes MYC protein conformation [51], by catalyzing conversion of Pro-63 MYC to a trans conformation [52]. PP2A-B56α is activated by isomerization of Pro-63 MYC, and functions in proteasome-mediated degradation of pThr-58 MYC via E3 ubiquitin ligases [53, 54]. The phospho-binding domain of Pin1 confers recognition of phosphorylated MYC sites [54], and the interaction of Pin1 with MYC can be affected by phosphorylation of both Thr-58 and Ser-62 [55], where Thr-58 is more critical than Ser-62 for Pin1 binding to MYC [64]. Additionally, Pin1 stabilizes the cis conformation of pSer-62-MYC, which prevents PP2A-B56a from dephosphorylating Ser-62 [56].

##### 2.2.1.6 E3 Ubiquitin Ligases

Various E3 ubiquitin ligases, such as Skp2, Fbw7, HectH9, and TRUSS, contribute to maintenance of MYC hemostasis via ubiquitin-ligase degradation [57]. Fbw7 isoforms are encoded through alternative splicing and regulate MYC turnover [58]. As a mediator of MYC turnover, Fbw7 targets p-Thr 58 and p-Ser sites in MBI [59]. On dephosphorylation of Ser-62 by PP2A-B56a, Fbw7 E3 ligase recognizes pThr-58 and recruits the 26S proteasome to degrade MYC proteins [60].

##### 2.2.1.7 Axis inhibition protein 1 (Axin1)

Axin1, a scaffold protein, recruits MYC, Pin1, PP2A-B56α, and GSK-3β to form a tetramer, which undergoes ubiquitin-mediated degradation [41]. Chromatin immuno-precipitation assays, to detect interaction between Axin1 and MYC transcriptional activity, demonstrated that Fbw7, Pin1, PP2A-B56α, and GSK-3β, as well as parts of the 26S proteasome, participated in the interaction [61].

##### 2.2.1.8 E3 Ubiquitin Ligases (Skp2 and Fwb7)

Fwb7 and Skp2 are major E3 ligases involved in inducing two ubiquitin-proteasomal degradation pathways to suppress MYC expression [62, 63]. Romo1, a mitochondrial modulator of reactive oxygen species (ROS) release into the cytoplasm, can translocate Fwb7 and Skp2 into the cytoplasm to induce cytoplasmic MYC degradation [64]. Skp2 interacts via conserved functional motifs: a helix-loop-helix-leucine zipper and MBII of MYC [34]. These interactions promote MYC degradation during G1 to S phase transition, independent of phosphorylation status [65]; however, Skp2 has contradictory effects on MYC transcription, also acting as a cofactor to stimulate the *MYC* promoter and increase its transcription [46]. Hence, Skp2 is vital in maintaining physiological levels of MYC.

##### 2.2.1.9 Proteasome activator subunit 3 (PSME3, also known as REGγ)

REGγ was first identified as Ki antigen, which functions to suppress MYC TF activity by interacting with its C-terminus, leading to degradation [66]. REGγ knockdown significantly increases MYC stability and affects MYC-mediated gene expression and cell growth [66].

##### 2.2.1.10 p53-induced RING-H2 (Pirh2)

The ubiquitin ligase activity of Pirh2 is critical in tumorigenesis, through mediating MYC poly-ubiquitination and proteolysis [67]. Skp2 can form a complex with MBII (MYC C-terminal domain), and Pirh2 (both the C- and N-termini) [67]. MYC protein expression is significantly increased in Pirh2-knockdown human RKO cells or Pirh2-deficient mouse NIH3T3 cells, demonstrating that Skp2 and Pirh2 are essential for regulating MYC turnover in tumorigenesis [68].

##### 2.2.1.11 Promyelocytic Leukemia Zinc Finger (PLZF)

PLZF is a TF involved in cellular proliferation and differentiation, thereby mediating developmental processes [69], and significantly represses MYC transcription and phosphorylation by binding to the *MYC* promoter and its MB sequences [70]. PLZF modulates AKT/MAPK signaling to decrease MYC phosphorylation at Ser-62 [70]. MYC dephosphorylation increases its stability and prevents ubiquitin-proteasomal degradation via E3 ubiquitin ligases [71].

##### 2.2.1.12 microRNAs (miRNAs)

miRNAs, which comprise 21-25 nucleotide molecules, have recently emerged as potential oncogene\tumor suppressors that inhibit MYC expression in cancerous or precancerous cells [72]. miRNAs target specific untranslated sequences within the genetic code, to induce excision or gene silencing [73]. During DNA damage, *miR-34c* is triggered and targets MYC to induce gene silencing [74], which inhibits DNA synthesis and repair and controls cell proliferation [75]. This pathway is a potential treatment target in MYC-induced cancer, in combination with other anti-cancer drugs.

### 2.3 Mechanisms of c-MYC-mediated Gene Regulation

#### 2.3.1 MYC Transcription and Regulation

*MYC* mRNA-levels are regulated by numerous signaling pathways, TFs, and chromatin components [76]. *MYC* family genes have several promoters, including P0, P1, P2, and P3, along with various initiation regions [41]. There are four Ca^2+^-regulated nuclear factors in activated T cell proteins, NFAT1/2/3/4, that were discovered in T lymphocytes [77]. NFAT1/2 bind to upstream DNA promoter elements to stimulate *MYC* transcription [78]. Further, *MYC* expression is upregulated in mouse T lymphocytes by Ca^2+/^calcineurin/NFAT1, where NFAT1 acts as a TF binding to the distal *MYC* promoter to increase *MYC* transcription [49].

MYC stabilization and activity are also influenced by post-translational modifications [61]. MYC regulation and cofactor recruitment are dependent on MBI and MBII respectively [62]. The MBI region contains two highly conserved phosphorylation sites, Ser-62 and Thr-58, which have important roles in stabilization of all mammalian MYC isoforms [79].

#### 2.3.2 Equilibrium Points in MYC Transcription and Regulation

MYC-MAX forms a dimeric complex with E-boxes, [80, 81], which are enriched in the promoters of genes involved in cell proliferation regulation [82-84], and MYC/MAX binding to E-boxes has several roles in gene regulation [82, 84].

Although MYC-MAX appears to regulate gene expression primarily through binding to E-box sequences in target gene regulatory regions, MYC can also interact with non-E-box DNA sequences [23, 85]; for example, there are non-canonical-E-boxes in ribosomal genes, which MYC binds to promote transcription [86, 87]. Additionally, MYC may function independently of MAX in some situations [88]. In a study of neuroblastoma, N-MYC was found to participate independently in regulation of *p53* (*p53 tumor suppressor homolog*) transcription [88]. Hence, under certain circumstances, MYC can induce biological functions without dimerization with MAX. Further studies to explore this mechanism are warranted.

MYC can selectively regulate transcription from its target genes, through the equilibrium among MYC, MAX, MAD/MXD (MNT), and MIZ1 binding to promoter regions [89]. In malignancy cell models, MYC proteins are usually overexpressed, favoring formation of MYC/MAX dimeric complexes, which promote transcription, leading to cell hyperproliferation, and thereby inducing tumorigenesis [89]; however, in the presence of MIZ1, tumor-favoring conditions are restored to equilibrium, as MIZ1 forms a ternary complex with MYC/MAX, which represses MYC-activated genes and suppresses hyperproliferation [90]. Further, MAD can function as a competitive inhibitor of MYC, as it has the same DNA binding domain as MAX and competes for this common target, reducing the rate of MYC-MAX binding [89]. MYC cannot activate transcription independently; hence, MYC-induced tumorigenesis-associated genes can be downregulated by MAD expression to diminish tumor cell hyperproliferation [89]. The equilibrium of MYC/MAX, MIZ1/MYC/MAX, and MAD (MNT)/MAX complexes represents a potential therapeutic target for guiding or controlling MYC target gene expression regulation and influence on the cell cycle (Fig. 1C). When the balance is favored toward MYC/MAX, the cell will be driven by MYC overexpression and undergo tumorigenesis; conversely, when the balance is reversed or equilibrium reached, MYC expression is controlled to maintain normal cell cycle regulation. Continued refinement of these MYC transcriptional repression models represents a promising future research avenue.

## 3. Functional Roles of MYC in Cancer Cells

Targeting MYC is among the highest priorities for cancer therapeutics. Dysregulated MYC expression is generally associated with poor patient prognosis [91]. Numerous *in vitro* and *in vivo* tests have shown that MYC is among the most potent oncogenes in inducing transformed cell phenotypes [92, 93]. Interestingly, the first observation of MYC upregulation causing neoplastic transformation only involved certain cell lines and was attributed to those cells having acquired other mutations that made them permissive [93]. Despite its prominent role in cancer pathogenesis, MYC overexpression alone cannot mediate cellular proliferation or neoplastic transformation [94, 95], rather, MYC overexpression affects normal cells in a highly destructive manner, resulting in cell death, senescence, and/or proliferative arrest [95, 96].

**Figure 2.**
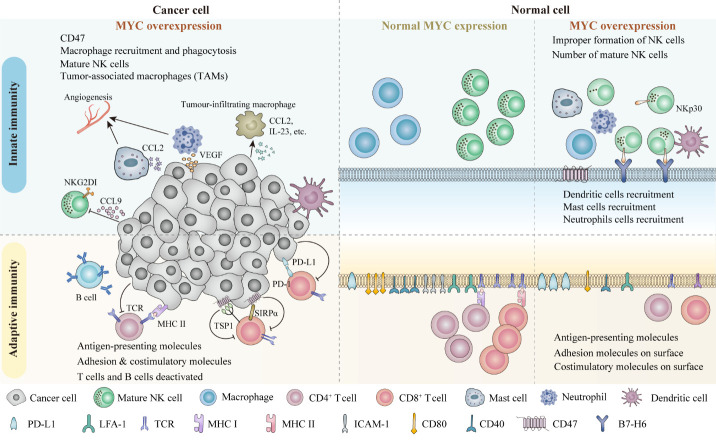
**Multiple roles and representative mechanisms of action of MYC towards the adaptive and innate immune responses in cancers**. MYC is a critical factor in manipulating immune-related event. The above left figure shows the influences of both the innate and adaptive immune cell responses in MYC-driven tumor. MYC overexpressed tumor cell is associated with reduced T cell-mediated anti-tumor immune responses; impaired macrophage and NK cells anti-tumor immune responses; recruited mast and neutrophil cells to promote angiogenesis; and alteration of the antigen-presenting molecules and the adhesion molecules on the cells surface. The middle section is a zoom in figure of the cells with normal MYC expression. This figure provides an overview of the immune cells and the adhesion & costimulatory molecules present in the MYC regulated condition. The right section of the figure has demonstrated the immune responses towards malignant cells with MYC overexpression. The MHC class I and class II molecules, adhesion and costimulatory molecules are decreased. MYC overexpression is associated with reduced cytotoxic T cell responses. MYC overexpression premotes the programmed death-ligand 1 (PD-L1) expression, which mediates the cells to escape from the T cells recognition. Also, MYC overexpression reduces NK cell amounts, and induces the expression of immune checkpoint CD47, preventing macrophage (Mph)-induced phagocytosis.

Tumorigenesis is initiated by MYC in cooperation with numerous other oncogenic events [97]. In MYC-induced tumor formation screens, many oncogenes were found to cooperate with MYC [96]. MYC frequently synergizes with genetic abnormalities which disrupt cell-cycle checkpoints and affect cell proliferation, cell death, senescence, and/or malignant transformation, due escalation of antiapoptotic events, such as BCL-2 upregulation, p53 downregulation, and p19ARF reduction, among others [98-100], indicating that normal cells possess a wide range of intrinsic tumor suppression mechanisms to control MYC expression and prevent malignancy [101].

Overall, MYC acts as a tumor-initiating gene, influencing cell cycle progression, as well as immune responses, via tumor-intrinsic epigenetic mechanisms [102, 103]. Angiogenesis can be controlled through modulation of the tumor microenvironment (TME) [104], which may interfere with various anti-cancer genetic events to induce tumorigenesis [35-37]. Hence, the local microenvironment has a critical role in MYC-induced tumor formation.

Herein, we summarize and discuss the capacities of MYC in various cancer processes, including immune responses, metabolism, the cell cycle, apoptosis, autophagy, pyroptosis, metastasis, angiogenesis, multidrug resistance (MDR), and intestinal flora.

### 3.1 MYC and Cancer Immune Responses

Several immune pathways are regulated by MYC to attack or eliminate carcinomas, such as prostate, colon, lung, and breast cancers [105, 106]. MYC overexpression can facilitate the escape of cancerous or pre-cancerous cells from anti-tumor immune cell recognition [105]; this escape iteration from immune responses is a hallmark of cancer [105]. The influences of overexpressed MYC on adaptive and innate immune cells are illustrated in Fig. 2.

#### 3.1.1 Impact of MYC Overexpression on Innate Immunity

The missing-self hypothesis states that natural killer (NK) cell activity targets infectious and tumor cells when human leukocyte antigen (HLA) class I is missing from, or downregulated on, the cell surface [107]. Inhibition of HLA class I expression by MYC can boost NK cell function [108, 109]; however, HLA class I downregulation can also cause NK cells to attack self-derived cells [105]. MYC is overexpressed in a murine T cell carcinoma model, leading to reduced NK cell maturation [110]. Further, *MYC* mRNA levels are positively correlated with the expression of B7-H6, a NKp30 ligand that activates NK cell-mediated degranulation [111, 112].

MYC also represses innate immunity through its effect in promoting tumor-associated macrophages (TAMs) [112]. MYC overexpression induces an immunosuppressive TME through the release of chemokines, growth factors, and inflammatory cytokines, as well as promoting activation of anti-immune checkpoint proteins, such as CD47, HIF, and TRVP1, in tumor cells to suppress M1 and M2 macrophage activation [112-114].

Inflammation contributes to cancer and *in vivo* MYC activation stimulates mast cells and neutrophils, which rapidly migrate to the tumor site, inducing various cytokines and growth factors, such as vascular endothelial growth factor (VEGF) and chemokine (C-C motif) ligand 2 (CCL2), that facilitate angiogenesis and promote tumor metastasis [115, 116].

#### 3.1.2 Impact of MYC Overexpression on Adaptive Immunity

MYC overexpression downregulates antigen-presenting and costimulatory molecules on tumor cells, enabling them to avoid immune system recognition; hence, MYC overexpression disrupts physical interactions between T cells and cancerous/precancerous cells with negative consequences [105].

##### 3.1.2.1 MYC- induced Antigen Presentation

Immune surveillance is generally compromised in tumors, due to their high MYC expression levels, which prevent recognition by cytotoxic T cells [109, 117]. MYC-overexpressing MDA-MB-231 and DU-145 B cell lines exhibit lower HLA class I expression, which influences binding between cytotoxic T and B cells; lung and colon carcers also exhibit similar characteristics [107, 117, 118].

MYC expression also impacts the generation of B cell-HLA class II; human leukocyte antigen DM (HLA-DM) is the HLA class II expression editor, and HLA-DA is controlled by MYC expression levels [118]. In a preclinical Burkitt lymphoma model with constitutive MYC expression, antigen-presentation to CD4^+^ T helper/regulator cells was reduced, due to downregulation of HLA-DM expression[118, 119]. Further, MYC knockdown led to recovery of HLA-DM levels and partial restoration of antigen-presentation to CD4^+^ T cells [118].

##### 3.1.2.2 MYC Overexpression with Adhesion and Costimulatory Molecules

T cell activation and recruitment rely on adhesion molecules, including vascular cell adhesion molecule-1 (VCAM-1) and intracellular adhesion molecules (ICAMs) [120]. T cell migration and activation is triggered by ICAM-1 binding to lymphocyte function-associated antigen-1 (LFA-1) on T cell receptors [120]; however, these intracellular adhesion molecule complex pathways are downregulated by MYC expression [121]. Furthermore, tumor necrosis factors (TNFs) responsible for mediating T lymphocyte recognition, cellular connectivity, and B cell adhesion, including TNF-α, lymphotoxin-α (TNFSF1), and lymphotoxin-β, are also repressed by MYC [119].

Co-factor signals are crucial in immune system modulation. On activation, costimulatory molecules produce secondary signals that enhance T cell responses [122, 123]. Deactivating MYC decreases CD40 molecules (CD40) expression in conditional knockout cell lines [122], where CD40 is a costimulatory protein on antigen-presenting B cells that interacts with CD40L (CD154) on T helper (T_H_) cells to recruit TNFs for immune response activation [123]. Additionally, MYC inhibits CD80 expression, preventing its binding to T cell co-costimulatory proteins (CD28/B7), thereby restricting T cell proliferation and activation [124]. Nevertheless, the mechanism underlying the relationship between MYC overexpression and CD40 levels remains elusive. No association between higher MYC protein levels and increased *CD40* transcription was observed in patients with diffuse large B cell lymphoma; however, MYC overexpression decreases levels of the co-stimulatory genes, *TNF receptor superfamily member 4 (TNFRSF4*) and *forkhead box P3 (FoxP3*), in regulatory T cells (Tregs) [125].

##### 3.1.2.3 MYC induced Programmed Death-Ligand 1 (PD-L1)-mediated T cell tolerance.

PD-L1 (also referred as B7-H1) is a transmembrane protein ligand of PD-1, encoded by the *CD274 molecule* (*CD274*) gene. A major role of MYC involves binding with PD-1, which transmits inhibitory signals to T cells to regulate antigen-specific T cell proliferation [126]. MYC overexpression induces PD-L1 mRNA and protein expression in prostate, breast, colon, and lung cancers [16, 127-129]; *PD-L1* mRNA expression is decreased proportionally to MYC protein inactivation [128]. In a murine carcinoma model, MYC bound the *PD-L1* promoter to induce its expression, resulting in increased PD-L1 protein levels [112]. Analogous results were obtained in human MDA-MB-231, DU145, MCF-7, and HCA-7 cell lines treated with MYC inhibitors [130, 131]. Nevertheless, some researchers have proposed that MYC expression has no role in regulation of PD-L1 expression [131]. Mice treated with JQ1 (a MYC inhibitor) had reduced PD-L1 expression relative to controls, while little PD-L1 downregulation was found in mice treated with MYC-specific shRNA [131]; hence, PD-L1 downregulation may be caused by other effects of JQ1 inhibitors, rather than MYC inactivation.

In conclusion, the mechanism involved in MYC regulation of PD-L1 remains ambiguous. Published studies have used various approaches to investigate the complex interactions between MYC and PD-L1 expression levels, revealing different roles of various factors in mediating PD-L1 expression across a number of carcinomas.

#### 3.1.3. MYC in Inflammation-mediated Neoplastic Transformation

Inflammation related or/and driven carcinogenesis is an area of intense cancer research. This type of neoplastic transformation involves chronic and systematic inflammation, as well as carcinogenesis. MYC, as a signature tumor driver [132-134], is a critical player in malignant transformation from inflammation to cancer.

In colorectal cancer (CRC), malignant transformation invariably involves inflammatory gastrointestinal disorders and cancerous colonic disease. Accordingly, therapeutic approaches are generally divided into anti-inflammatory and anti-cancer aspects [135]. In a nuclear factor-erythroid 2 (NF-E2) -related factor 2 (NRF2) knockout Apc^min/+^ mouse model, increased c-MYC expression in intestinal tissue is accompanied by high proliferating cell nuclear antigen (PNCA) levels, which promote intestinal carcinogenesis and adenomas, due to intestinal crypt cell proliferation [136]. c-MYC protein synthesis is increased specifically in intestinal epithelial cells, independently of Wnt-APC-β-catenin signaling, in response to overexpression of CD98, which is a crucial transmembrane glycoprotein that exhibits oncogenic activity in inflammation-associated intestinal tumorigenesis [137]. Further, the phosphorylated Smad3L (pSmad3L)/c-MYC oncogenic signaling pathway promotes ulcerative colitis-associated neoplastic progression [138].

Mast cells are inflammatory cells necessary for macroscopic expansion of pancreatic islet tumors, and rapid recruitment of mast cells is triggered by MYC activation [115, 139]. In human pancreatic ductal epithelial cells, increased MYC expression and protein synthesis were induced by AT-rich interaction domain 1A (ARID1A) knockdown, and pancreas-specific ARID1A loss could also elicit inflammation and pancreatic intraepithelial neoplasia formation in mice [140].

c-MYC has a comparatively indirect role in hepatocarcinogenesis, interacting with tumor necrosis factor receptor-associated factor 6 (TRAF6) to promote hepatocarcinogenesis through TRAF6/HDAC3/c-MYC signaling, which is primed in hepatitis B virus-transgenic mice [141]. Downregulated c-MYC expression is consistent with tumor growth suppression on blocking of the CCL2/CCR2 axis, which is a potential target for patients with hepatocellular carcinoma and chronic hepatic inflammation [142].

TNF-related apoptosis-inducing ligand receptor (TRAIL-R) deficiency in mice affects MYC-driven lymphomagenesis, highlighting its potential role in susceptibility to inflammation-driven carcinogenesis [143]. Transformation of high-grade B-cell lymphoma to gastric diffuse large B-cell lymphoma involves a MYC-dependent malignant transformation pathway [144]. In inflamed environments with related inflammatory signals, decreased TRAF6 expression increases MYC transcriptional activity, promoting leukemia transformation [145]. In large granular lymphocyte leukemia initiated by overexpression of IL-15, c-MYC contributes to up-regulation of Aurora kinases and *miR-29b* suppression [146].

**Figure 3.**
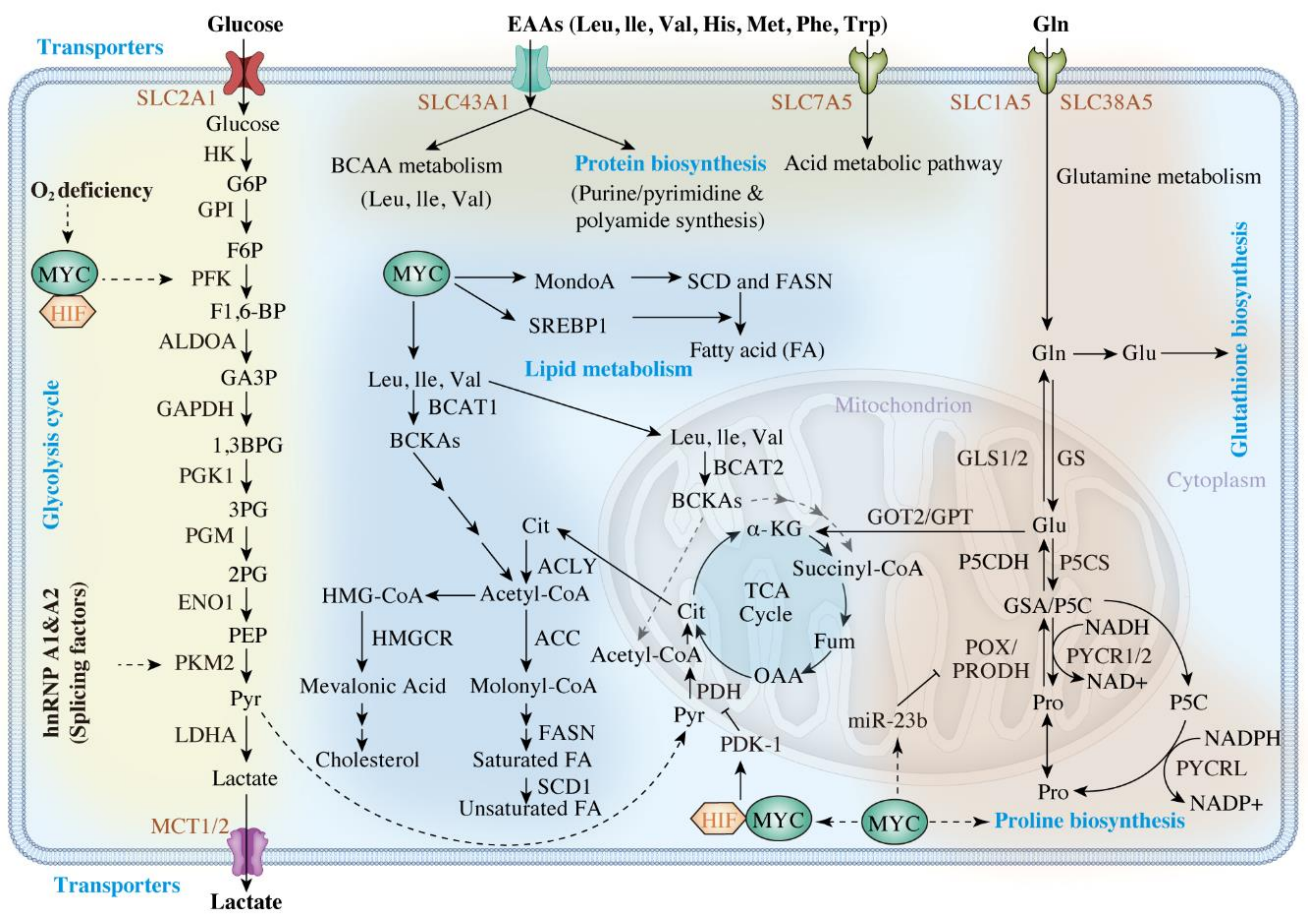
**The role of MYC in metabolism alteration and its consequences. A wide range of glycolytic enzymes are upregulated by MYC, which supports glucose metabolism**. MYC promotes the expression of SLC1A5 and SLC38A5 to increase the glutamine uptake and promotes the glutaminolysis-related enzymes (GS/GLS1/2) to elicit glutamine addiction. MYC promotes proline anabolism *via* increasing PYCR1/2 and represses its catabolism *via* decreasing POX/PRODH expression. Enzymes labelled with red arrows are upregulated by MYC, and those labelled with blue arrows are suppressed by MYC. Also, MYC activates critical transporters, SLC7A5, SLC43A1, and SLC1A5, to promote essential amino acid transport. BCAT1, which catalyzes the decomposition of branched amino acids, is a downstream target of MYC. MYC coordinates glucose, glutamine, and essential amino acid metabolism to promote fatty acid biosynthesis. Enzymes labelled in red are upregulated by MYC. α-KG α-ketoglutarate, ALDOA aldolase A, 1,3BPG 1,3-bisphosphoglycerate, Cit citrate, ENO enolase, F1,6-BP fructose 1,6-bisphosphate, F6P fructose 6-phosphate, GA3P glyceraldehyde-3-phosphate, GAPDH glyceraldehyde-3-phosphate dehydrogenase, Gln glutamine, GLS glutaminase, Glu glutamate, GOT2 glutamate oxaloacetate transaminase, G6P glucose-6-phosphate, GPI phosphoglucose isomerase, GPT glutamine pyruvate transaminase, GS glutamine synthetase, GSA glutamic-γ-semialdehyde, HK hexokinase, LDHA lactate dehydrogenase A, Mal malate, MCT monocarboxylate transporter, P5C Δ1-pyyroline-5-carboxylate, P5CDH P5C dehydrogenase, P5CS P5C synthase, PDH pyruvate dehydrogenase, PEP phosphoenolpyruvate, PFK phosphofructokinase, PG phosphoglycerate, PGK phosphoglycerate kinase, PGM phosphoglucomutase, PKM2 pyruvate kinase M2, POX/PRODH proline oxidase/dehydrogenase, Pro proline, PYCR P5C reductase, Pyr pyruvate, SLC solute carrier family, Suc succinate, TCA tricarboxylic acid, ACC acetyl-coA carboxylase, ACLY ATP citrate lyase, BCAA branched-chain amino acid BCAT branched-chain aminotransferase, BCKA branched-chain α-keto acid, FA fatty acid, FASN fatty acid synthase, Fum fumarate, His histidine, HMG-CoA 3-hydroxy-3-methylglutaryl-CoA, HMGCR 3-hydroxy-3-methyl-glutaryl-coenzyme A reductase, Ile isoleucine, KMO kynurenine-3-monooxygenase, Kyn kynurenine, KYNU kynureninase, Leu leucine, Met methionine, OAA oxaloacetate, Phe phenylalanine, SCD stearoyl-CoA desaturase, Thr threonine, Trp tryptophan, Val valine.

Moreover, c-MYC exerts metabolic-related modulation effects. Chronic inflammatory signaling, such as activation of the STAT3/c-MYC axis, may elicit gradual metabolic reprogramming, represented by elevation of key metabolic enzymes involved in promoting progression from chronic colitis to CRC [147]. Promotion of inflammation and tumorigenesis by stromal fibroblasts through metabolic reprogramming is mediated by mTORC1/c-MYC signaling [148]. Additionally, during epigenetic blockade of neoplastic transformation by the bromodomain and extra-terminal (BET) domain protein inhibitor, JQ-1, c-MYC levels decreased rapidly in mouse skin epidermal JB6 P+ cells [149]. Some proinflammatory cytokines, including interferon γ (IFN-γ) and TNF-α, synergistically induce tumorigenesis via NF-κB-mediated c-MYC activation in ovariectomized mice [150].

### 3.2. MYC and Cancer Metabolism

#### 3.2.1 MYC and the Warburg Effect (Glycolytic Metabolism) in Cancer

Aerobic glycolysis is important in creating the TME and inducing the Warburg effect [151], a metabolic adaptation characterized by enormous glucose uptake, glycolysis, and lactic acid generation in oxygen-sufficient environments, to support aerobic respiration [151]. Through this process, a TME containing numerous anabolic precursors, with sufficient energy to promote cell mutagenesis and support tumor cell community formation emerges, particularly under hypoxic conditions.

MYC regulates aerobic glycolysis by binding to the classical E-box consensus sequence in glycolytic genes [152] ; for example, SLC2A1 is a glucose transporter, which can enhance glucose uptake efficiency in the presence of MYC (Fig. 3) [153]. Immunoprecipitation assays indicated that MYC binds to E-box regions at the *hexokinase 2 (HK2*), *lactate dehydrogenase A (LDHA), and enolase 1 (ENO1)* loci, which are highly conserved across eukaryotic species [152]. Lactate can be transported away from cancer cells through MYC-activated MCT1 (SLC16A1 solute carrier family 16 member 1 aliase) and MCT2 (SLC16A7 solute carrier family 16 member 7 aliase*)* channels [154]. Furthermore, *glyceraldehyde-3-phosphate dehydrogenase (GAPDH)* and *TPI* are both regulated by MYC using alternative mechanisms, since non-canonical E-boxes are present in their upstream promoters [152]. Glycolytic genes are activated by MYC via both transcription and alternative splicing [155]. Splicing factors promote processes favoring glycolysis, as expression of the pyruvate kinase, PKM2 (an enzyme involved in aerobic glycolysis), exceeds that of PKM1 (which mediates oxidative phosphorylation) in response to MYC activation of the protein coding genes, *heterogeneous nuclear ribonucleoprotein A1 (hnRNPA1)* and *heterogeneous nuclear ribonucleoprotein A2 (hnRNPA2)* [155].

Besides MYC, a second TF, hypoxia-inducible factor-1α (HIF-1α), controls tumor cell glycolysis under oxygen deficient conditions [156]. In adenocarcinoma of the glandular prostrate and lobular breast milk duct, glycolysis-related genes, including *HK2* and *pyruvate dehydrogenase kinase 1 (PDK1)*, are activated by MYC and HIF-1α under hypoxic conditions [156, 157], whereas MYCN and HIF-1α cooperate to mediate anaerobic glycolysis in other malignancies. These findings indicate interactions between MYC oncoproteins and HIF-1α in cancer metabolism [156, 157].

#### 3.2.2 MYC and Amino Acid Metabolism in Cancer

##### 3.2.2.1 Essential Amino Acids (EAAs)

Cancer cells can either synthesize amino acids or recruit extracellular amino acids through protein-gated channels [158]. MYC expression triggers alterations in amino acid metabolism [158, 159]. EAAs are a subset of amino acids described as essential in humans, because they cannot be independently produced and must be derived from alternative sources or the extracellular environment [160]. These EAAs serve as subunits for cellular component anabolism and as ligands that induce signaling pathways [160, 161]. The EAA transporters, SLC7A5/SLC43A1, induce MYC protein synthesis and transcription of downstream targets, which can disrupt several de novo metabolic processes, including glycolysis, ammonolysis, and lipogenesis (Fig. 3) [161]. For example, tryptophan metabolism can be altered in various ways in cancer cells. Like SLC7A5, SLC1A5, and arylformamidase, MYC regulates the kynurenine pathway in colonic cells, increasing conversion of tryptophan to kynurenine [162]. Increasing kynurenine levels promotes cancer cell proliferation and migration and provides an immune barrier to tumors [163]. Moreover, MYC can enhance glutamine uptake efficiency through activating the glutamine transporters, SLC1A5 and SLC38A5, thus promoting glutamine catabolism [164]. In PC3 prostate cancer (PCa) models, MYC elevates glutaminolysis by indirectly enhancing translation of glutaminase 1 (*GLS1*), via inhibiting the *GLS1* repressors, *miR-23a/b* [158]. MYC regulation of glutamine catabolism can have varying outcomes, according to the different metabolic requirements of diverse cancer types. Besides glutamine catabolism, MYC also regulates glutamine synthetase (GS) to control glutamine anabolism [165]. GS catalyzes nitrate reduction of ammonia to glutamate during glutamine formation and MYC demethylates the GS promoter by TET3 upregulation, which upregulates thymine DNA glycosylase expression [165]. Such aberrant expression upregulates various cellular components, including glutamine and amino-acid transporters, to support tumor outgrowth, as observed in a human PCa model [165, 166]. As both GS and GLS1 are transiently expressed in different tumor cell subcellular compartments, MYC can activate both reactions simultaneously in an individual cell; glutaminolysis occurs in mitochondria, whereas glutamine synthesis primarily takes place in the cytosol [167].

##### 3.2.2.2 Non-essential Amino Acids (NEAAs)

Biosynthetic and degradation pathways of several NEAAs also appear to be regulated by MYC; for example, comprehensive clinical data indicate that a subset of neoplastic cancers induced by MYC are associated with tumorigenic proline metabolism [168]. MYC upregulates P5C reductase (PYCR) and P5C synthase (P5CS), influencing the conversion of glutamine to proline [168, 169] , which can resolve conditions involving proline deficiency and maintain homeostasis [170]. Furthermore, MYC can suppress proline dehydrogenase/proline oxidase (PRODH/POX) expression via upregulation of *miR-23b*, thereby diminishing proline catabolism and endoplasmic reticulum (ER) stress [168, 169]. Moreover, MYC induces tumorigenic processes through stimulating serine biosynthesis by activating 3-phosphoglycerate dehydrogenase (PHGDH) and phosphoserine amino-transferase (PSAT1) [171] , which stimulates glutathione (GSH) production and nucleotide biosynthesis [159]. Along with upregulating serine anabolism, MYC promotes serine catabolism and glycine synthesis through serine hydroxymethyltransferase 2 (SHMT2) [172] . In contrast, growth defects induced by MYC-deficiency in MYC-knockdown murine fibroblast models were moderately recovered by SHMT2 [173] ; however, it had no effect on constitutive MYC expression in colorectal adenomagenesis [174]. Hence, the relationship between MYC and SHMT2 remains unclear, and the mechanisms may depend on microenvironmental factors. In addition to tumorigenesis, MYC also facilitates serine/glycine biosynthesis, along with HIF-1α and ATF4, which can integrate the anaerobic glucose metabolic cycle in tumor cells [174, 175].

#### 3.2.3 MYC and Lipid Metabolism in Cancer

Tumorigenesis requires acceleration of lipid synthesis for cell membrane proliferation, and MYC is key to regulation of fatty acid synthesis and oxidation, cholesterol generation, and liposomal signal molecule transportation [176].

##### 3.2.3.1 Fatty Acid Production

MYC stimulates fatty acid synthesis in prostate, colon, and breast cancers [177-180], and MYC upregulates several TCA cycle genes to produce the fatty acid precursor, citrate (Fig. 3) [178, 181]. Further, MYC activates production of acetyl-CoA carboxylase (ACACA), ATP citrate lyase (ACC), stearoyl-CoA desaturase (SCD), and fatty acid synthase (FASN), which are involved in fatty acid synthesis [176, 182]. Furthermore, MYC upregulates the TF, MondoA, to increase the transcript levels of *SCD* and *FASN*, which are vital in promoting saturated fatty acid synthesis [183]. Further, MYC associates with the element-binding protein, SREBP1, to promote fatty acid synthesis [182].

##### 3.2.3.2 Cholesterol Metabolism

Malignant cells overexpressing MYC upregulate 3-hydroxy-3-methylglutaryl-coenzyme A reductase (HMGCR) to promote cholesterol synthesis [184]. In MYC-driven tumor models, MYC activation and phosphorylation are HMGCR-dependent, leading to a feedback loop that triggers tumorigenesis and cancer metabolic alterations [185].

##### 3.2.3.3 Fatty Acid Oxidation (FAO)

In cancer metabolism, MYC promotes FAO, a process occurring in mitochondria, to facilitate ATP synthesis via oxidation of fatty acids in most eukaryotic cells [176, 186]. This process was demonstrated in a MYCN knockdown neuroblastoma model, in which decreased expression of several FAO-related enzymes (ETFA, HADHA, and HADHB) was detected [186]. MYCN inhibition also disrupts the mitochondrial respiratory chain, interfering with FAO redox function [186]. Similar phenomena were also detected in MYC-induced breast cancer (TNBC) and PCa (PC-3) models, where oncogenic levels of MYC induced both CD36 and CPT1A/CPT2 expression to stimulate fatty acid uptake into the mitochondrial inner membrane, where it undergoes complete oxidation [187-189]. In calcium (Ca^2+^) signaling, MYC activates FAO by promoting AMP-activated kinase (AMPK) activity in a Ca^2+^-CAMKK2-dependent manner [188]. In contrast, MYC was recently reported to inhibit FAO via downregulation of the same pathway, including HADHA, HADHB, ACADL, and ACADVL, contradicting an earlier publication [176]. These findings suggest that MYC can either accelerate or reduce FAO function, according to the cellular microenvironment.

#### 3.2.4 MYC-dependent Regulation of Metabolism Contributes to the Tumor Immune Microenvironment

##### 3.2.4.1 Glycolysis

MYC-induced metabolic reprogramming correlates with the emergence of the tumor immune microenvironment. Cancers often exhibit high glucose consumption, due to higher levels of MYC, corresponding with increases in MYC-regulated glycolytic enzymes (HK2, TPI, ENO1, PKM2, and LDHA) [190]. Aberrant MYC expression leads to increased levels of LDHA, which converts pyruvate into lactate to acidify the extracellular environment [191, 192]. This hinders NK and cytotoxic T cell targeting of tumor cells and recruits Tregs, repressing immune response initiation and favoring an M2-like phenotype of TAMs [191-195]. Escalating glucose uptake in the TME results in glucose-deficiency in T cell metabolic pathways, which negatively impacts T cell surveillance, downregulates IFN-γ production, and amplifies PD-1 levels [196, 197] , thus contributing to impaired antitumor immunity. T cells released in acidic conditions secrete fewer cytokines (such as TNF-α, IFN-γ, and IL-2) and express higher CTLA-4 levels [198].

##### 3.2.4.2 Glutamine Transport

MYC overexpression in cancer is attributable to the fact that glutamine is necessary for cell growth and genetic events. In addition to enhancing glutamine transport into cells, MYC upregulates glutamine transporter expression and GLS enzymes through promoter demethylation, facilitating glutamine-to-glutamate conversion [199, 200]. Additionally, the glutamine-to-glutamate pathway increases competition for glutamine metabolites in tumors, causing naïve T cells to differentiate into immune-suppressive Tregs, and impairing NK cell function [196, 197].

##### 3.2.4.3 Metabolic Regulation

A lipid metabolic process regulated by TRPV1, a transmembrane cation channel, gated by heat, and low tissue pH, activated by MYC provides energy to tumors with high energy demand, which are characterized by poor vascular circulation and deficient lymphatic drainage [201, 202]. This leads to a lack of critical metabolites and a surplus of waste products in the TME, inducing tumorigenic stress and representing a pre-stage of malignant metastasis [201].

### 3.3. MYC and the Cell Cycle

#### 3.3.1. MYC Expression and the Cell Cycle

MYC is an established modulator of cell cycle progression and mediator of cell proliferation rates. One important function of MYC in the cell cycle is promotion of entry into S phase, as demonstrated in a MYC-deficient rat fibroblast model, which has a longer G1 phase than that of wild-type cells [203]. HectH9 mediates MYC-mediated entry into the cell cycle [204]; in a human HectH9-deficient tumor cell model, cells are paused at G1 phase [204]. MYC stabilization is mediated through two signaling pathways: PI3K-AKT and Raf-MEK-ERK [33]. ERK and GSK-3β play opposing roles in preventing or triggering MYC degradation during the early (ERK) and late (GSK-3β) phases, respectively, by phosphorylating Ser-26 and Thr-58 [205].

Abnormal MYC levels push cells to enter S-phase and undergo immortal cell division, without the need for growth factor stimulation [206]. Schuhmacher et al. provided evidence of a steady increase in cell proliferation rate in a model with increased MYC levels [207, 208]. Further, Wang and colleagues demonstrated that depletion or silencing of MYC in 23 cell lines, including healthy and tumor cells, using MYC antisense oligonucleotides, led to cessation of G0/G1 or G2/M cell cycle transitions [209]. The MXD protein can prevent cell cycle progression by antagonizing MYC-mediated target gene transcription [62]; MXD shares a similar DNA binding domain with MYC and competes with MYC to bind with MAX [210]. MAX/MXD dimerization prohibits MYC mediated transcription, leading to cell cycle arrest. Blocking cyclin B1 (CCNB1) upregulation can inhibit cell cycle arrest by MXD1, causing starved cells to release HIF-1α, which arrests the cell cycle by counteracting MYC expression under hypoxic conditions [210].

#### 3.3.2 Biological Factors Involved in MYC Cell Cycle Regulation

##### 3.3.2.1 Cyclin-dependent kinases (CDKs)

Many CDK genes, including *cyclin dependent kinase 4 (CDK4)* and c*yclin dependent kinase 6 (CDK6)*, are upregulated by MYC [211]; however, its effects on *cyclin dependent kinase 2 (CDK2)* are controversial. In one study, the authors reported increased CDK2 mRNA and protein levels on MYC overexpression, but another investigation showed that the gene plays in a different role [212]. ChIP assays indicated that Ras and cyclin C interact with MYC to bind the *cyclin dependent kinase 1 (CDK1)* promoter and augment CDK1 expression. As MYC promotes the Cdk-activating kinases (CAKs) transcriptional activity, which phosphorylates the activation segment (CDK T-loop) and increases CDK levels [213, 214]. Furthermore, MYC restrains CDK inhibitory effects through induction of either *miR-221* effects on Wee1 or activation of Cdc25 (cell division cycle 25) phosphatase [213, 214]. *miR-221* also targets *p27, p57, and Rb* mRNAs, hindering their CDK inhibitory properties [213-215].

##### 3.3.2.2 Cyclins

MYC also regulates cyclin expression; however, there are controversies regarding the role of MYC in regulating cyclin D1. Expression of cyclin D1 can be increased, suppressed, or unaffected by MYC, depending on the cell type [216]. Additionally, MYC induces cyclin D2 expression by recruiting TRRAP [217] and induces cyclin E1 by direct regulation of E2F TF expression [218]. Researchers identified MYC target genes by serial analysis of gene expression and found that the cell cycle mediators, cyclin B, cyclin E binding protein 1, and Cdc2-L1, control MYC-induced transition between G1, S, and G2 phases [219].

##### 3.3.2.3 CDK Inhibitory (CKI) Proteins

CKI proteins, such as INK4 and CIP/KIP family molecules, can repress CDKs [220, 221]. cyclin dependent kinase 4/6 (CDK4/6) activities are inhibited by binding of INK4 family proteins, which interferes with their kinase activity [220-222]. Also, the INK4 family proteins, p15 and p16, prevent Rb phosphorylation and arrest cell cycle progression by preventing selective removal of the INK4 CDK inhibitor, p27, from cyclin D-CDK4/6 and its redistribution to cyclin E-CDK2 [223].

##### 3.3.2.4 ADP-ribosylation factor (ARF)

The *ARF* gene maps to human chromosome 9p21 [224], is upregulated by MYC to inhibit cell cycle progression, and mediates apoptosis, with or without the p53 pathway [224]. On ARF activation, MDM2 proto-oncoprotein is released from p53, which stabilizes p53 and activates p21 induction to trigger apoptosis [225]. ARF inhibits MYC transactivation, thereby preventing its hyperproliferative and transformative effects; however, ARF cannot prevent MYC-induced apoptosis [226, 227], possibly because other MYC-associated apoptotic genes can also induce apoptosis [226].

##### 3.3.2.5 RB transcriptional corepressor (Rb) Hypophosphorylation

MYC controls cell cycle progression by both upregulating specific genes and inhibiting negative cell cycle regulators [228, 229]. MYC binds to E-boxes in the E2F promoter and induces transactivation of a set of genes related to G1 to S-phase transition [230]. E2F activity depends on Rb phosphorylation level [228]; hypophosphorylated Rb binds E2F and suppresses its expression to disrupt cell cycle progression [228]. To overcome Rb hypo-phosphorylation, MYC induces cyclin/CDK upregulation via various mechanisms and signaling pathways, including gene expression induction or regulation by phosphorylation and dephosphorylation [228]. Elevation of CDK proteins can conditionally overcome Rb hypophosphorylation [228]. Further, MYC stimulates *miR-221* induction, which reduces Rb expression at the mRNA and protein levels, and prevents recurrence of Rb hypophosphorylation, to restrain cell proliferation [214, 231].

##### 3.3.2.6 p15

MYC can also inhibit the activity of negative regulators of the cell cycle [228]. G1 phase arrest is mediated by TGF-β-induced p21, which can be inhibited by AP4 transactivation through inhibition of MYC signaling [232]. Treatment of lung epithelial cells with TGF-β leads to rapid reduction of MYC and expression of p15, while exogenous MYC spontaneously enters cells to recover TGF-β-induced p15 levels to background levels [233]. Following TGF-β treatment, MIZ-1 exhibits high-affinity binding in the vicinity of the p15 promoter, stimulating *p15* transcription [234, 235]. SP1 and SMAD also interact with MYC to pause p15 expression. Additionally, on interacting with MYC and following replacement of their coactivators, SP1 can act as both a transcriptional activator and a repressor [234-237]. MYC forms an inhibitory complex with SMAD and SP1 that represses *p15* gene expression on exposure to TGF-β [237].

##### 3.3.2.7 p21

MYC controls p21 by various mechanisms, which prevent p53-induced apoptosis and override p21 regulation by p53 [238]. MYC counteracts DNA damage by regulating p21 and GADD45 production in response to p53-induced p21 [239-241]. Cdc2 kinase activity is inhibited by GADD45 by reducing Cyclin B1 nuclear localization [242]. MYC/Miz-1 dimerization is among mechanisms that directly or indirectly impair p21 expression [243] , while p21 induction by MYC inhibits KDM5B and TFAP2C formation of a ternary complex [244]. MYC-induced transcription regulators, such as AP4 and *miR-17-92*, also inhibit p21 induction [245].

##### 3.3.2.8 p27

The antagonistic relationship between p27 and MYC expression is established [246]. MYC downregulates p27 at both the transcriptional and post-transcriptional levels, recruits factors that bind to initiator element (Inr) in the p27 promoter, and downregulates FoxO3a expression, which is an essential factor in mediating p27 upregulation [246]. Upregulation of the MYC-dependent miRNAs, *miR-221* and *miR-222*, inhibits p27 post-transcriptional activity [215, 231]. MYC can counteract p27 expression and circumvent G1/S transition arrest in various ways. On Rb phosphorylation, MYC-mediated E2F TFs can activate S phase-related genes and downregulate p27, whereas MYC upregulates cyclin E transcription, which enhances the efficiency of cyclin E p27 redistribution from the cyclin D/CDK4/6 complex [247]. Additionally, the ubiquitin ligase, Skp1-Cullin-1-F-box (SCF), containing Skp2, is elevated by MYC, and recognizes and degrades p27 on cyclin E (induced by MYC) via phosphorylation of Thr-187 [248, 249].

##### 3.3.2.9 DNA Replication and Mitosis Proteins

MYC influences numerous genes involved in DNA replication and mitosis [250]. Initiation and elongation of DNA replication is mediated by CTD1, as well as MCM proteins (MCM3, MCM4, MCM5, and MCM6). Additionally, MYC increases replication origin activity by interacting with pre-replication complexes [211, 251-253]. Furthermore, MYC extends the anaphase stage by upregulating anaphase-promoting complex/cyclosome (APC/C), which degrades the mediators of metaphase-anaphase transition, cyclin B1 and securin [211, 254]. Unlike APC/C, MYC represses the securin gene, *PTTG1* [211]. Further, mitotic arrest deficient 2 (MAD2) and Bub1-related kinase1 (BubR1) expression are elevated in response to MYC overexpression, and mitotic arrest results in extended anaphase [255]. Furthermore, cells expressing low levels of MYC exhibit fewer apoptotic events than those overexpressing MYC [256], while MYC overexpression results in increased anomalous polyploidy, accentuating chromosomal instability via the presence of micronucleus amplifications [256]. Although normal mitosis occurs regardless of MYC levels, its duration and spindle structure formation are controlled by the amount of MYC present. Cells with high MYC levels have a wider equatorial plate, due to shorter spindle length. This delays chromosome alignment at metaphase and leads to late anaphase induction, causing mitotic cycle arrest. Cells overexpressing MYC also exhibit accelerated nuclear envelope breakdown. MYC also controls the mitotic cycle by influencing mitosis-related events, including centriole production, kinetochore assembly, proteolysis, and cytokinesis [256].

##### 3.3.2.10 miRNAs

MYC induces miRNAs that inhibit negative cell cycle regulators [257]. MYC-dependent activities are regulated by miRNAs that functionally interact with Let-7, of which *miR-34a* represses CDK4/6, E2Fs, and cyclin E2 expression levels; *miR-15a/16-1* regulates CDK6 and E2F3; and cyclin D2/E2 are suppressed by *miR-26a* [258, 259].

##### 3.3.2.11 H19

LncRNA H19, a MYC-induced molecule, forms a positive feedback loop with MYC expression [260] and is extensively transcribed under aberrant MYC expression conditions, leading to Rb silencing and escalation of cell proliferation [261]. LncRNA H19 also strengthens binding between MYC and specific cell cycle gene promoters, to control cell cycle transition via MYC induction [261].

### 3.4 MYC and Apoptosis

MYC is established to mediate apoptosis with its partner MAX [262]. Aberrant MYC expression in combination with antiproliferative stress/apoptotic signals, makes cells more fragile and vulnerable to apoptosis [262].

#### 3.4.1 MYC-induced Apoptosis Pathways

**Figure 4.**
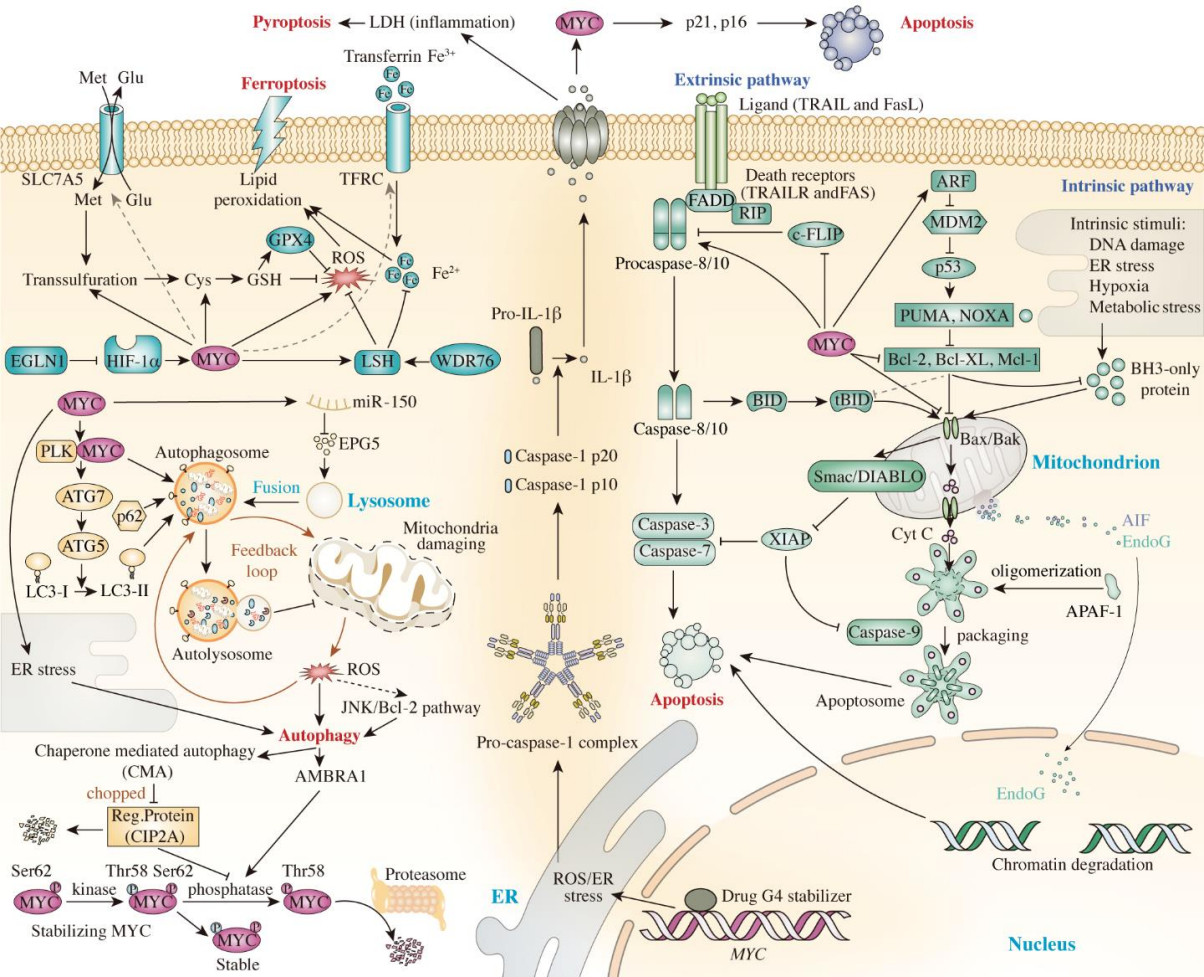
**The roles of MYC act in mediating cell death pathways and their mechanisms**. APAF-1: Apoptotic Protease Activating Factor-1, ARF: ADP ribosylation factor; ATG7: Autophagy Related 7; Bcl-2: B-cell lymphoma 2 protein; Bcl-XL: B-cell lymphoma-extra large; BID: BH3 interacting-domain death agonist; c-FlIP: Cellular FLICE (FADD-like IL-1β-converting enzyme)-inhibitory protein; Cys: Cysteine; DIABLO: Direct Inhibitor of Apoptosis-Binding protein with LOw pI; EGLN1: Egl-9 Family Hypoxia Inducible Factor 1; ER stress: Endoplasmic Reticulum Stress; FADD: Fas Associated Via Death Domain; G4-stabilizer: G4-quadruplex-stabilizer; Glu: Glutamic acid; GPX4: Glutathione peroxidase 4; GSH: glutathione; HIF-1α: Hypoxia Inducible Factor 1 Subunit Alpha; LC3: Microtubule-associated protein 1A/1B-light chain 3; LSH: lymphoid-specific helicase; Mcl-1: Myeloid cell leukemia 1; MDM2: murine double minute 2 homolog; Met: Methionine; PLK: Polo Like Kinase 1; PUMA: p53 upregulated modulator of apoptosis; RIP: Receptor-interacting protein; ROS: reactive oxygen species; Ser62: Serine62; Smac: Second mitochondria-derived activator of caspase; SQSTM1 : sequestosome 1; tBID: truncated BH3 interacting-domain death agonist; Thr58: Threonine58; WDR76: WD Repeat Domain 76; XIAP: X-Linked Inhibitor of Apoptosis.

MYC-induced apoptosis generally occurs in two ways: intrinsically (mitochondrial) or extrinsically (extracellular) (Fig. 4). The intrinsic pathway usually triggers the apoptotic cascade when cells are experiencing DNA damage, oxidative stress, or ER stress [263]. Consequently, apoptosis-inducing factors and cytochrome c (Cytc) are released into the cytosol, facilitating apoptosome formation, which activates pro-caspase molecules [262]. On cleavage and reformation of the apoptosome complex, caspase-3/7/9 are activated explosively, resulting in apoptosis [264]. The BCL-2 protein family is a vital mediator of apoptosis and can be categorized into three different subfamilies based on their functions: (1) the anti-apoptotic family, (2) the BH3 pro-apoptotic family, and (3) the pore-forming family. BCL-2 associated X, apoptosis regulator (BAX) and BCL-2 antagonist/killer (BAK) belong to the pore-forming family, which mediate channel formation in the outer mitochondrial membrane, allowing Cytc release into the cytosol [265]. BCL-XL and BCL-2 are anti-apoptotic proteins that prevent BAX and BAK from binding, thus limiting mitochondrial permeability and preventing Cytc export [266]. Thus, a balance between anti-apoptotic and pro-apoptotic molecule expression regulates Cytc secretion from mitochondria; if the expression is skewed, the equilibrium is disrupted, and expression progresses toward the favored side.

Extrinsic apoptosis pathways are triggered in the extracellular space by binding of death ligands to programmed-death receptors on the cell surface [267]. There are several groups of death receptors, including tumor necrosis factor receptor (TNFR), Fas, and TRAILR1 and 2, among others [267]. Death receptors contain 80 amino-acid cytoplasmic death domains, which induce apoptotic signaling to trigger an apoptosis cascade [267]. On ligation of death receptors, a death-inducing signaling complex is formed, comprising the adaptor molecule, Fas-associated death domain protein (FADD) , the c-FLIP initiator, and the inactive precursors, procaspase-8/10 [268, 269]. Caspase-8 activation occurs via stimulation of FADD signaling and homodimerization and procaspase-8 cleavage by autocatalysis [268, 269]. Caspases, such as caspase 3 and caspase 7, facilitate cell death, by cleaving and activating the active form of caspase-8 [270]. Additionally, BID is cleaved by caspase-8, generating truncated BID (tBID), which allows Cytc release from mitochondria via open MOMP channels [270]. BID mediates transition between the intrinsic and extrinsic apoptosis pathways [271, 272]. The master anti-apoptotic regulator, cFLIP, controls death receptor-mediated cell death by binding to FADD, rather than procaspase-8, thereby inhibiting caspase-8-FADD interaction [273].

#### 3.4.2 Apoptosis Factors and MYC

##### 3.4.2.1 Cytc

Apoptosis is triggered when MYC induces Cytc release from mitochondria [274], and MYC-dependent apoptosis is mediated by its transcriptional target, BAX. It has been proposed that BAX upregulation is induced or controlled indirectly by MYC [275]. The apoptosis response relies on BAX and BAK, and activating MYC alone is insufficient to induce apoptosis; thus, cells lacking BAX and BAK are less vulnerable to apoptosis induction, regardless of MYC levels [275, 276]. Overexpression of BCL-XL can inhibit MYC-induced BAK activation by blocking its conformationally activated form [275, 276]. BCL-XL is essential for apoptosis termination through inhibiting BAK activation [276]. There is evidence that MYC inhibits BCL-2 and BCL-XL through the mediator, BIM, whose promoter is bound by MYC to upregulate its transcription, facilitating proper BAX and BAK function, and allowing MOMP to release Cytc into the cytosol and induce apoptosis [277].

##### 3.4.2.2 Death Receptor-Ligand Systems

MYC can trigger extrinsic apoptosis through stimulation of cell surface receptors that respond to death ligands, such as TNF-α, Fas, or TRAIL [278]. Further, serine/threonine kinases can be activated by stimuli from activated cells to trigger apoptosis [279].

##### 3.4.2.3 FADD and Caspase-8

FADD and caspase-8 contribute significantly to apoptosis when MYC promotes expression of receptor-interactive protein (RIP) [280]. RIP and MYC are synergistic, in that caspase-8 and FAD, which have inhibitory effects on *c-FLIP* transcription, promote the chain activation of procaspases into functional caspases, followed by apoptosis [280]. MYC is prevented from triggering apoptosis when c-FLIP expression is moderate or ectopically high [280]. Direct or indirect caspase-8 increase occurs on post-translational modification of MYC [281], and MYC also increases FasL expression, which contributes to apoptosis induction [282].

##### 3.4.2.4 p53

MYC can induce apoptosis in several ways by interacting with p53, which regulates multiple proapoptotic genes involved in either the intrinsic pathway, the extrinsic pathway, or both [283]. In the presence of stable p53, apoptosis is accelerated through proapoptotic protein upregulation, while anti-apoptotic protein expression is reduced [284]. In contrast, p53 is controlled by the negative regulator, MDM2 E3 ligase, which maintains p53 at low levels by continuous ubiquitin-proteasomal degradation [285]. ARF upregulation occurs as a result of MYC aberrations, inhibiting MDM2 degradation, and thereby inducing apoptosis [286]. Absence of both p53 and ARF may attenuate MYC-related apoptosis [270, 286]; however, some research groups have proposed that MYC may induce an alternative apoptosis pathway, that does not require ARF or p53 [286-288].

##### 3.4.2.5 ROS

ROS is crucial in cell signaling and homeostasis maintenance [288]. Apoptosis can be triggered by ROS-induced phenomena, such as oxidative stress, ER disruption, and mitochondrial dysfunction [288]. During ectopic expression of MYC and E2F-1, NF-kB activity is notably inhibited, as is the negative regulator of ROS, superoxide dismutase (SOD) [288]. Excessive ROS results in programmed cell death [289]. ODC is a rate-determining enzyme involved in converting ornithine into putrescine, whose activity is elevated by MYC to increase polyamine production and degradation [290]. When polyamines accumulate beyond levels that can be utilized by polyamine oxidase, they are converted to ROS, ultimately inducing apoptosis [290].

##### 3.4.2.6 Forkhead box O3A (FoxO3a)

FoxO3a is a member of the FoxO gene family and an essential modulator mediating MYC stability and mitochondrial gene expression [291]. Besides dimerizing with MAX and downregulating transcription of MYC-mediated target genes, FoxO3a can also disrupt MYC translation by generating miRNAs that bind its mRNA [291, 292]. Moreover, MYC maintains a negative feedback loop with FoxO3a, which can replace FoxO3a and override downstream effectors, such as GADD45 and PUMA, thus suppressing FoxO3a expression [293, 294]. FoxO3a activation also reduces MYC-induced apoptosis. FoxO3a mitigates ROS generation as is a byproduct of mitochondrial metabolism and leads to apoptosis, by sequestering superoxide dismutase (SOD2) and catalase, protecting cells from elevated ROS-related stress damage [295]. Additionally, nuclear mitochondrial genes are regulated via the MYC-dependent FoxO3a pathway, which affects mitochondrial function and reduces cellular ROS levels [296, 297]. Interactions among MYC, FoxO3a, and nuclear-mitochondrial associated genes appear to be vital for regulation of MYC and ROS.

##### 3.4.2.7 Cell division cycle 25A(Cdc25A)

MYC and Cdc25A cooperate to promote apoptosis by directly targeting transcriptional activity [298]. The MYC/MAX heterodimer can promote Cdc25A expression, increasing its mRNA and protein levels, through binding to its promoter [299, 300]. Pim-1 mediates the apoptosis-inducing effects of MYC and Cdc25A by phosphorylating both proteins and stabilizing their conformation [300]. Hence MYC, Cdc25A, and Pim-1 have crucial interacting roles in triggering programmed cell death.

### 3.5. MYC and Autophagy

#### 3.5.1 MYC-dependent Regulation of Autophagy in Cancer Cells

##### 3.5.1.1 Autophagic Progress

In cells, organelles and granules are often digested via autophagy, which wraps proteins and organelle fragments, engulfs them in double-membrane-bound autophagosomes, and subsequently degrades them within fused autophagosomes and lysosomes [301, 302]. Genome-wide RNA sequencing by Toh and colleagues demonstrated that MYC participates in early autophagosome formation mediated by the JNK-BCL-2 pathway [301]. MYC-mediated autophagosome regulation controls release of the autophagy signaling receptor, p62, and MYC inhibition results in defective autophagosome formation and reduced autophagy substrate delivery [301] ; these findings illustrate the importance of MYC modulation in regulating autophagic processes, particularly in restraining autophagy.

##### 3.5.1.2 Autophagosome-lysosome Fusion

Induction of aberrant autophagy by ectopic MYC expression may contribute to development of non-small cell lung cancer (NSCLC) [303]. In NSCLC, *miR-150* is strongly associated with ectopic levels of MYC, and *miR-150* overexpression results in abnormal autophagic flux, with an increase in autophagosomes and a decrease in autolysosomes [303]. The decrease of autolysosomes may be attributable to repression of the autophagosome maturation gene, *EPG5* (ectopic P-granules 5 autophagy tethering factor), by *miR-150*, preventing autophagosome-lysosome fusion and triggering tumorigenesis [303]. Reduction of autolysosome formation limits autophagy, preventing proper degradation of damaged mitochondria which accumulate in cells [303]. Thus, A549 and H1299 NSCLC cells overexpressing *miR-150* secrete excessive ROS, while maintaining cell integrity [303].

##### 3.5.1.3 MYC Stabilization

MYC and PLK1 are major drivers of tumorigenesis, enhancing cancer cell growth and proliferation via autophagy [304] and increased PLK1 levels are associated with poor cancer prognosis. When MYC is knocked down, autophagy-related protein 7 (Atg7) and hallmarks of autophagosome formation, LC3-II and LC3-I, are markedly reduced, leading to defective autolysosomal degradation [304]. Similar effects have been observed in cells with PLK1 knocked down, which show significantly decreased LC3-II, LC3-I, and Atg5 expression, with concurrent SQSTM1 accumulation and autolysosomal pathway impairment [304]. Further, PLK1 is involved in MYC protein stabilization, and its inhibition leads to notably decreased MYC expression [304]. Significant tumor regression was detected in a mouse xenograft model treated with the PLK1 inhibitor, BI2536, compared with untreated controls [304], supporting a combined effect of PLK1 and MYC in tumorigenesis.

Ambra1 is a tumor suppressor scaffold protein that promotes MYC destabilization and degradation via dephosphorylating at pSer-62 [305]. Ambra1 also participates in autophagy signaling and its deficiency leads to tumor hyperproliferation through MYC hyperphosphorylation, thereby causing tumorigenesis [305]. In addition, Ambra1 also promotes PP2A phosphatase dephosphorylation of MYC, destabilizing MYC and inhibiting cancer cell proliferation [305]. Hence, PLK1 and Ambra1 are potential therapeutic targets for treatments aimed at modulating MYC stabilization.

##### 3.5.1.4 Endoplasmic Reticulum Stress

MYC is associated with the ER stress/autophagy pathway. Hart and colleagues showed that the cell lines, P493-6 (human lymphoblast) and MEF (mouse fibroblast), undergo autophagic transformation and tumor growth on induction of ER stress [306]. Specifically, the unfolded protein response (UPR) can increase cell survival by inducing autophagy via activation of PERK/eIF2α/ATF4 [306]. MYC-mediated autophagy is reduced, and tumorigenesis remarkably diminished by PERK inhibition, whereas apoptosis occurs due to autophagy inhibition [306]. Reduced autophagy was evident on blocking ER stress, which led to reversion of protein synthesis to normal levels [306]. Thus, therapeutic targets involving UPR, ER stress, and autophagy may emerge.

#### 3.5.2 Autophagy and Mitophagy Regulation by MYC and Chaperones

##### 3.5.2.1 Cancerous Inhibitor of PP2A (CIP2A)

The tumor growth-promoting pathway chaperone-mediated autophagy (CMA) correlates with MYC expression [307]. Kon and colleagues discovered that CMA had a tumor-suppressive effect on MEF cells, promoting proteasomal degradation and inhibiting MYC oncogenic activity [307]. CMA destabilization of MYC occurs through control of CIP2A degradation, which reduces phosphorylase levels, inhibiting Ser-62 dephosphorylation and proteasomal degradation of MYC [308]. Hence, CIP2A is a regulatory protein, and blocking CMA results in reduced CIP2A degradation, which has potential to prevent cancer development [308].

##### 3.5.2.2 miRNA

Various studies have established associations between MYC and mitophagy, which involves selective destruction of the mitochondrial membrane via autophagy as a result of long-term stress or damage [309]. Treatment of cells with hydrogen peroxide for a prolonged period induces mitophagy and decreases nuclear GSK-3β levels, reducing MYC phosphorylation, and elevating *miR-106b-93-25* [310]. In response to elevated *miR-106b-93-25* levels, the miRNA cluster inhibits mitophagy substrate proteins, restoring the cellular energy balance by blocking excessive mitophagy pathway activation, which can trigger bioenergetic collapse and cell lethality [310, 311]. Overall, these findings imply that miRNA interaction with mitophagy substrate proteins functions to maintain cell survival and assist in mitophagy regulation.

##### 3.5.2.3 Bax interacting factor 1 (Bif-1)

The membrane protein, Bif-1, is an important connection linking mitophagy, apoptosis, and autophagy [312]. When autophagosomes form during mitophagy, Bif-1 is necessary to maintain chromosome stability, while *Bif-1* haploinsufficiency suppresses mitophagy and accelerates MYC-induced tumorigenesis by expanding mitochondrial mass and promoting the malignant state [312]. On loss of Bif-1, the inability of MYC-induced tumors to clear damaged mitochondria by autophagy or mitophagy suppression has been suggested to cause chromosomal instability, resulting from oxidative stress and DNA damage [312].

### 3.6. MYC and Pyroptosis

Pyroptosis involves activation of inflammatory responses as part of a programmed cell death pathway [313, 314]. Despite sharing some similarities with apoptosis, pyroptosis uniquely involves activation of caspase 1. MYC stabilization of G-quadruplex (G4) nucleic acid secondary structure inhibits MYC function and induces ER stress and pyroptosis [315]. Gaikwad and colleagues defined D089 as a specific MYC-G4 ligand and demonstrated that it binds specifically to DNA G4 within the myeloma promoter to inhibit MYC transcription, likely causing cell death by one of two mechanisms: cell senescence or caspase-1-dependent pyroptosis [315]. Caspase-1-dependent pyroptosis is characterized by formation of pyroptosomes, which are required to convert pro-caspase-1 into active p10 and p20 caspase-1 molecules and for release of interleukin 1β (IL-1β) and IL18 inflammatory cytokines into the cytoplasm [315]. Caspase-1 also engages in the cleavage of cell fragments to form pores, which permeabilize cell membranes, leading to IL-1β release, activating inflammatory proteases and cytokines that subsequentially trigger pyroptosis [315]. Furthermore, secretion of IL-1β (a by-product of MYC inhibition-induced pyroptosis) may upregulate MYC, thereby increasing the expression of senescence-associated factors, such as p21 and p16, inducing cell death via apoptosis, or promoting tumorigenesis, during which cells become immortal [316]. To conclude, pyroptosis, apoptosis, and cancer have context-dependent relationships. Thus, by examining the interactions between MYC and pyroptosis, it may be possible to design novel cancer treatments, particularly for apoptosis-resistant cancers that often escape apoptosis by overexpressing anti-apoptotic proteins.

### 3.7. MYC and Ferroptosis

Ferroptosis is a novel form of programmed cell death involving metabolic dysfunction that alters lipid metabolism and causes iron-dependent ROS production, as well as generating aberrant levels of iron transferrin, glutathione peroxidase 4 (GPX4), and p53, among other molecules [317-320]. MYC has complex roles in ferroptosis-related signaling.

#### 3.7.1 Lymphoid-specific helicase (LSH)

Egl nine homolog 1 (EGLN1) and MYC promote LSH through a pathway involving HIF-1α and the inhibitory effects of LSH are mediated by WDR76, to enhance the expression of genes involved in lipid metabolism [321]. Jiang et al. built a model to explain the inhibitory effects of LSH on ferroptosis and its influence on tumorigenesis, in which the effects of LSH on ferroptosis are mediated by regulation of a novel mechanism involving several metabolism-related genes. EGLN1 counteracts the effects of HIF-1α by preventing binding between c-MYC and HIF-1α, allowing c-MYC to bind the *LSH* promoter and upregulate its transcription [321]. Interaction between LSH and WDR76 elevates levels of lipid metabolism-associated genes, including *solute carrier 2* (SLC2)*, facilitated glucose transporter member 1* (*GLUT1*), *fatty acid desaturase 2* (*FADS2*), and *stearoyl-coenzyme A desaturase 1* (*SCD1*), among others [321] , thereby inhibiting cellular accumulation of iron and lipid ROS (crucial factors in ferroptosis), preventing ferroptosis and promoting tumorigenesis [321]. In contrast, c-MYC and LHS expression levels are reduced when EGLNs are inhibited and HIF-1α is induced [321]. In experiments, treatment with BAY inhibited EGLN, resulting in lack of EGLN1 and c-MYC engagement at the *LSH* promoter due to HIF-1α elevation, which can counteract both MYC and EGLN1 expression [321, 322]. Following CoCl_2_ treatment, which replaced BAY and liberated EGLN1, EGLN1 and c-MYC were recruited and bound to the *LSH* promoter, and LHS expression recovered [321, 322]. These results indicate that the c-MYC/EGLN1 axis can increase LSH expression, consistent with the authors’ hypothesis, whereas HIF-1α functions as a repressor of LSH expression that counteracts EGLN1 to prevent MYC binding to the *LHS* promoter [321-323]. Thus, double-gated regulation of LHS by MYC/EGLN and HIF-1α has potential to elevate intracellular ROS levels, which is a prerequisite for triggering ferroptosis and provides an alternative cell death pathway to kill apoptosis and autophagy resistant MYC-driven cancer cells.

#### 3.7.2 Consequences of MYC-driven Lipid Metabolic Alteration in Ferroptosis

On MYC-induced alteration of lipid metabolism, cellular cysteines are converted into glutathione, and the absence of cellular cysteine induces massive lipid peroxidation, which increases ROS levels [324]. Further, MYC enhances iron uptake through activation of the transferrin receptor gene, *transferrin receptor* (*TFRC*) gene, which causes ferroptosis [324]. MYC also elevates SLC7A5 expression, which imports methionine in exchange for glutamine, without cystine uptake [324]. Cellular cysteine is derived from the MYC-driven methionine cycle and transsulfuration, and feeds into glutathione biosynthesis [324, 325]. Under oncogenic ‘MYCN-high’ and cysteine-deprived conditions, cellular glutathione is crucial for preventing lipid ROS accumulation and avoiding ferroptosis [324, 326]. These findings imply that cysteine-dependent glutathione availability regulates the function of oncogenic MYC(N) in ferroptosis. Hence, a novel therapeutic approach could be developed based on the enzymes and antiporter proteins crucial to ferroptosis, representing novel opportunities for MYC-based therapeutic interventions.

Overall, mammalian cells are highly regulated by MYC-induced programmed cell death and specific mechanisms for inducing cell death by regulating MYC levels are beneficial. In this review, we cover several programmed cell death mechanisms (apoptosis, autophagy/mitophagy, pyroptosis, and ferroptosis), which could serve as alternative targets for treatment of resistant cancers, by reprogramming the cellular context to prevent tumorigenesis. Diverse methods targeting these pathways could be used in combination with one other and with classical cancer hallmark inhibitors to effectively eliminate or prevent malignant neoplasm progression and drug/pathway-resistant tumor development.

### 3.8 MYC and Cell Metastasis

#### 3.8.1 MYC and Cancer Cell Migration

##### 3.8.1.1 MYC Promotion of Cancer Cell Migration

###### 3.8.1.1.1 Actin Cytoskeleton

Various cancers are associated with deregulation of MYC family transcriptional regulators, which contributes to malignant transformation through regulating biomass accumulation and cell proliferation [327]. Anderson et al. demonstrated that MYC cleavage by the endogenous proteasome to generate a truncated form, MYC-nick, mediates cancer cell migration and stimulates metastasis [327]. MYC-nick upregulates expression of fascin, an actin-bundling protein, as well as activating Cdc42, a GTPase subunit of Rho, to reconfigure the actin cytoskeleton [327]. MYC is elevated in a MYC-induced model of human CRC and migrating cells at the invasive front of the tumors expressed high levels of both Cdc42 and fascin [328]. Filopodia are structures that function to direct cell migration, and upregulation of Cdc42 and fascin results in filopodia formation [327, 328] , consistent with the results of several studies suggesting that abnormal cytoskeleton structure and fascin upregulation drive motility and metastatic behavior, representing an alternative function of MYC [329-332].

###### 3.8.1.1.2 Epithelial-to-mesenchymal Transition (EMT)

MYC can also promote cellular invasion and migration via upregulation of EMT-associated genes [333]. MYC induces several mesenchymal TFs, including OPN, SNAIL (through TGF-β activation), and LGALS1, to promote cell migration [334-337]. Further, MYC forms a transcriptional complex with SKp2, MIZI, and p300 to induce tumor migration and metastasis via RhoA activation [333]. Zhao and colleagues constructed a murine lung cancer model using HepG2 cells with RNAi-silenced c-MYC [338], and demonstrated that MYC expression was dramatically decreased, while transwell chamber cell migration assays showed significantly reduced migration of cells with c-MYC silenced, relative to controls [338]. Hence, MYC may contribute to cancer cell migration.

##### 3.8.1.2 MYC Suppresses Cancer Cell Migration

Contrary to the role of MYC in promoting tumor cell migration, one study found that MYC can suppress cell migration. Ma and colleagues identified MYC as a negative regulator that impeded the migratory and invasive capacity mediated by Ras and Lgl and decreased expression of the JNK signaling target, matrix metalloproteinase (MMP-1) [339], thereby interfering with tumor migration and metastasis [339]. Further c-MYC can increase apoptosis, reduce cell motility, and inhibit cell migration. Alfano performed a transcriptomic analysis and found that MYC suppresses the expression of urokinase (uPA) and urokinase receptor (uPAR), which are crucial mediators of cell migration, adhesion, and growth mechanisms, thereby influencing cell migration [340]. MYC-induced downregulation of uPA and uPAR causes significant rearrangement of cancer cell cytoskeletal architecture (cells become rounder and compact and grow in tighter clusters), which impedes cancer cell migration by impairing their ability to invade the extracellular matrix (ECM) [340]. Furthermore, MYC activates the caspase3/7 cascade to initiate a series of cellular events that trigger p53 induction and p21 targeting, stimulating apoptosis and preventing cancer cell migration, suggesting that MYC suppresses cancer cell migration by stimulating apoptosis and disrupting expression of genes involved in cell migration [340]. Overall, these studies demonstrate that MYC has contradictory effects on cell migration, which are likely context-dependent.

#### 3.8.2 MYC in Cancer Cell Invasion

MYC also functions in cell invasion and is associated with several prognostic signatures involved in tumor invasion and metastatic growth.

##### 3.8.2.1 Ezrin

Ezrin is associated with c-MYC induction of PCa in the presence of androgens [341]. Aberrant androgen levels cause ezrin phosphorylation, thereby regulating downstream AKT and GSK-3β signaling [341], which induces MYC protein synthesis and prohibits its degradation [341]. MYC overexpression leads to increased binding of the *ezrin* promoter and enhances its transcription [341] , thereby inducing upregulation of downstream genes, such as *RhoA*/*Cdc42* and *Akt*, among others [341], with important roles in mediating cell invasion. In summary, there is a positive feedback loop between c-MYC and ezrin, which acts with androgens to influence PCa cell tumorigenesis.

##### 3.8.2.2 Gastric Carcinogenesis long non-coding RNA1 (GClnc1)

MYC also regulates expression of *GClnc1*, a long non-coding RNA (lncRNA) that significantly promotes J82 and 5637 bladder cancer cell invasion and metastasis by elevating MYC activity [342], consistent with reports that *GClnc1* overexpression in bladder cancer can promote cell migration and invasiveness [343, 344]. *GClnc1* promotes cancer progression by partially activating MYC; *MYC* mRNA levels were significantly increased on GClnc1 overexpression and significantly decreased after *GClnc1* silencing [342]; hence, MYC and *GClnc1* levels are positively correlated. Further, MYC activation overrides *GClnc1* inhibition, restoring cell invasiveness, while *GClnc1* up-regulation activates MYC, resulting in bladder cancer progression [342]. Hence, lncRNAs can modulate MYC activity to control cancer cell invasion, and the function of *GClnc1* in regulating MYC activity warrants further exploration.

##### 3.8.2.3 NDRG family member 2 (NDRG2)

Genes downstream of MYC have vital roles in suppressing cell invasion via reduction of matrix metalloproteinase (MMP)-2/9 activity [345]. N-MYC downstream-regulated gene 2 (NDRG2) is a candidate tumor-suppressor, while MMPs can cause cancer metastasis via ECM protein degradation and triggering cell invasion [346, 347]. Faraji and colleagues evaluated NDRG2 overexpression using gelatin zymography; pro- and active forms of MMP-2/9 were detected in the gel in the control group, which lacked NDRG2 expression, whereas cells overexpressing NDRG2 showed significant reductions in the pro-and active forms of MMP-2/9 as well as significantly reduced invasion compared with the control group [345]. The mechanism underlying NDRG2 inhibition of invasion is ambiguous. Nevertheless, there is increasing evidence supporting a role for NDRG2 as a tumor suppressor that reduces metastatic activity via MMP-2/9 [345, 347, 348]. Further research is required to explore how these pathways contribute to the effectiveness of NDRG2 in treating malignant tumors.

Overall, MYC represents a crucial biomarker for tumor invasion, since it interacts with various genes implicated in cell invasion. Research on MYC-related biomarkers is required to develop strategies to control cancer cell invasion in the future.

### 3.9 MYC and Angiogenesis

An ample blood supply is critical for tumor progression and maintenance, and blood vessel development is required to ensure that oxygen, nutrients, and growth factors can be delivered to cells [349]. In cancer progression, MYC is essential for angiogenesis, which promotes sprouting of new capillaries from preexisting vessels, to provide factors required for tumor growth [349, 350].

#### 3.9.1 HIF-1α

c-MYC can induce angiogenesis via HIF-1α [349] which participates in angiogenesis as an essential vascular factor contributing to TME formation [349, 350]. c-MYC overexpression can stimulate HIF-1α expression by preventing protein degradation [349]. In experiments assessing HIF-1α mRNA and protein in LoVo (colon epithelial cells) with c-MYC either overexpressed or knocked down, *HIF-1α* mRNA levels were similar in both groups, while HIF-1α protein was markedly increased and stabilized in the c-MYC overexpressing compared with the knockdown group, indicating that c-MYC does not influence HIF-1α transcription, but is rather involved in stabilizing HIF-1α protein [349]. MYC can also regulate expression of VEGF protein, an essential target of HIF-1α, which signals neovascular tissue (vessel) growth [350, 351]. MYC overexpression promotes *VEGF* transcription, leading to high levels of VEGF mRNA and protein [349, 350, 352]. Further, platelet-derived growth factor-B (PDGF-B) is a HIF-1α-responsive gene whose expression level modulates MYC expression and can cause aberrant neovascularization by generating inappropriate angiogenic signals [353]. c-MYC activation is regulated by PDGF-B, which promotes Src homology 2 domain-containing tyrosine phosphatase 2 (SHP-2) activity [353]. PDGF-B phosphorylates MYC Ser-62, stabilizing the protein and inhibiting proteasomal degradation [353], and leading to ectopic MYC expression and HIF-1α upregulation, forming a positive regulatory loop with PDGF-B [349, 353]. Constitutive PDGF-B expression increases SHP-2 levels, which enhances angiogenic signaling via the ERK pathway to induce aberrant neovascularization [353]. Thus, PDGF-B is at least partially responsible for cell proliferation and angiogenesis through activation of SHP-2/ERK/c-MYC [353]. Based on these findings, c-MYC and HIF-1α have been identified as potential therapeutic targets in colon cancer [354], which could theoretically lead to clinical trials targeting these factors to promote anti-tumor activity.

#### 3.9.2 Tumor Endothelial Marker 8 (TEM8)

N-MYC and TEM8 (an integrin-like cell-surface transmembrane protein), induce tumor endothelium outgrowth, contributing to the progression of several types of cancer, and are associated with cancer angiogenesis [355]. To determine if PCa angiogenesis can be induced by overexpression of N-MYC and TEM8, a tubule formation assay was conducted using human umbilical vein endothelial cells, and immuno-histochemistry analysis revealed that N-MYC and TEM8 expression levels were positively correlated in PCa tissue [355]. While N-MYC and TEM8 have established roles in promoting PCa progression, the underlying mechanism remains poorly understood; however, targeting the N-MYC/TEM8 pathway appears to be promising for treatment of PCa and TEM8 may be a useful indicator of treatment responses in patients with PCa [355]. Further research and a detailed investigation of the effects of N-MYC in mediating TEM8 expression in PCa are required prior to commencement of clinical trials.

In summary, due to the importance of MYC for coordinated expression of angiogenic factors required for tumor progression, disruption of MYC functions has potential to be effective for treating angiogenesis-dependent tumors.

### 3.10 MYC and Multidrug Resistance (MDR)

Cancer development of drug resistance is generally due to increased expression of membrane transporters, resulting in decreased intracellular concentrations of anticancer drugs as they efflux from cancer cells [356-358]. The effects of chemotherapy on cancer cells are hindered by MDR characteristics, which contribute to poor patient prognosis [357]. MYC upregulation is associated with multidrug refractory disease and contributes to MDR in patients with cancer [359]. Several representative proteins are associated with MYC-induced MDR, as outlined below.

#### 3.10.1 Nuclear Receptor Corepressor 2 (NCoR2)

Multiple myeloma cells express high levels of MYC, associated with downregulation of NCoR2 [360]. NCoR2 is a corepressor that targets various TFs involved in cancer growth and development [361]. In a CRISPR/cas9- NCoR2 knockout model, NCoR2 knockdown led to MYC upregulation [360]. Further, the inhibitory activities of histone deacetylases (HDACs), pomalidomide, and BET were significantly decreased in NCoR2-null/repressed cells, independent of Cereblon (CRBN), suggesting that the risk of MDR is related to high MYC expression [360]. MYC upregulation is mediated by the NCoR2-CD180 pathway and formation of the NCoR2-NuRD complex repressed CD180 expression in NCoR2 knockout cell lines, causing MYC upregulation, regardless of CRBN induction [360]. These findings indicate the presence of a novel drug resistance pathway independent of CRBN induction and suggest that NCoR2 expression may be a potential biomarker for study of immunomodulatory imide drug refractory disease and could be applied to regulate MYC expression to overcome MDR in cancer cells.

#### 3.10.2 Prostate Cancer-associated ncRNA Transcript 1 (PCAT-1)

PCAT-1 accelerates c-MYC-mediated PCa cell proliferation and is associated with MDR development in CRC [362], as well as disease progression [363, 364]. PCa cell proliferation is facilitated by PCAT-1 upregulation [364], while PCAT-1 inhibition reduces CRC cell metastasis and proliferation [362]. The effects of PCAT-1 on MDR development in CRC cells were investigated by applying 5-fluorouracil (5-Fu) to Caco-2 and HT-29 cells; cells with PCAT-1 knocked down showed significantly lower viability after 5-Fu treatment than parental PCAT-1-expressing control cells [362]. Further, PCAT-1 knockdown CRC cells exhibited dramatically reduced c-MYC production, correlated with reduced c-MYC-dependent invasiveness and drug resistance; however, overexpression of MYC in PCAT-1 knockout cells partially restored cell invasion and drug resistance, demonstrating that PCAT-1 is a regulator of the *MYC* gene and that c-MYC protein is fundamental in triggering PCAT-induced cancer cell aggression [362]. These findings demonstrate that PCAT-1 modulates cell invasiveness and drug resistance via regulating c-MYC expression.

#### 3.10.3 P-glycoprotein (P-gp)

c-MYC is frequently overexpressed in MDR variants, and its levels are positively correlated with the abundance of P-gp on cancer cell membranes [365]. The P-gp transporter and MDR-associated proteins, MRP1 and ATP binding cassette subfamily C member 1 (ABCC1), are particularly relevant to cancer chemotherapy, as are the breast cancer resistance proteins, BCRP and ABCG2, which are encoded by *GTPase-activating protein MDR1* (*MDR1*) genes [365]. P-gp functions differ depending on its physiological location; it maintains blood-brain barrier integrity, is involved in excreting drugs from the kidneys and liver into urine and bile, and pumps drugs absorbed in the intestine back into the lumen [366, 367]. P-gp has major roles in pharmacovigilance of drugs through its transporter efflux function. Elevated c-MYC expression induces P-gp activation, contributing to MDR development [368-370]. The mechanism of P-gp induction by MYC was elaborated using CHIP assays, which showed that intracellular c-MYC levels do not directly regulate P-gp expression, rather direct binding of c-MYC to the *small nucleolar RNA host gene 12 (SNHG12*) promoter was observed and shown to enhance its transcription [368]. In two transfected NK cell subclones (YTS and SNK-6 cells), a significant change in P-gp expression level was observed on SNHG12 regulation, which influenced cell sensitivity to cisplatin and paclitaxel (CDDP) [368, 371]. Experiments to verify the relationships among MYC, SNHG12, and P-gp showed that SNHG12 and MYC overexpression partially increase levels of P-gp and Ki67 (a gene that promotes cellular proliferation) in transfected YTS cells and promote cancer cell proliferation by desensitizing cells to CDDP; whereas P-gp expression and CDDP sensitivity were markedly reduced by knocking down SNHG12 in SNK-6 cells [368, 371]. Hence, SNHG12 protein exerts its biological function through posttranscriptional interactions with c-MYC, where SNHG12 mediates upregulation of P-gp activation. Furthermore, some intermediate factors induced by MYC overexpression, such as *miR-20a*, HIF-1α, and Nrf2, can upregulate P-gp levels [372-375]. Hence, the invention and development of drugs targeting intermediate components of MDR pathways, such as P-gp or factors induced by it, mediated by MYC overexpression is a potential area for research focus.

#### 3.10.4 Bromodomain PHD Finger TF (BPTF)

MYC interacts with BPTF to induce MDR in cancer cells through upregulation of ABC transporters [376]. BPTF is a cofactor that alters chromatin structure to increase transcription activation and recruitment of c-MYC to ABC-transporter promoters, elevating their expression and contributing to gemcitabine (drug) resistance [377, 378]. Drug efflux through ABC-transporters is responsible for developing drug resistance because it reduces the amount of drug available for absorption into a tumor [377, 378]. In Velasco’s research, a BPTF-inhibited mouse model was generated to examine the effect of BPTF on tumor cell proliferation, sensitivity to gemcitabine, and expression of ABC-transporters [376]. BPTF silencing impaired c-MYC recruitment and binding to the promoter of the *ABC-transporter (ABCC1*), thus impairing its transcriptional regulation [376]. Hence, inhibition of BPTF represses ABC-transporters, reducing gemcitabine efflux, and leading to its accumulation in cells, causing DNA damage and subsequently inducing programmed cell death [376, 379], demonstrating that combined BPTF-silencing and gemcitabine treatment can have complementary effects in treating MDR cancer [376]. Therefore, BPTF is an attractive potential therapeutic target (rather than direct targeting of MYC) to bypass mechanisms of drug resistance via regulation of ABC-transporter expression.

#### 3.10.5 AMP-activated protein kinase (AMPK)

The metabolic sensor and stress redox checkpoint, AMPK, is required for c-MYC-mediated survival under stress conditions, as AMPK has a tumor-protective role in MYC-driven cancer [380, 381]. Inhibition of the AMPK pathway leads to apoptosis in c-MYC-overexpressing cells, whereas AMPK activation prevents c-MYC-knockdown-mediated cell death by diminishing intracellular oxidative stress [382]. Further, intracellular levels of SirT1 and AMPK, which are essential factors in supporting MYC expression, can influence MDR characteristics during lung cancer treatment [380, 382]. NSCLC tumors are resistant to drugs such as cisplatin and doxorubicin under hypoxia and normal conditions due to SirT1 downregulation, since H1299 and A549 cells expressing SirT1 had significantly lower IC_50_ values for cisplatin and doxorubicin, relative to those with SirT1 knocked down, revealing that SirT1 overexpression enhances drug sensitivity in NSCLC cells, while its knockdown confers resistance to anticancer drugs [383, 384]. AMPK inactivation during hypoxia cooperates with SIRT1, leading tumors to develop drug resistance [383]. Assessment of AMPK activity *in vitro* demonstrated that it is activated by SirT1 via deacetylation and activation of LKB1 [383, 385]; however, under hypoxia, SirT1 reduced LKB1 expression and inactivated AMPK [383]. In this regard, SirT1 functions as a component of the AMPK pathway, where SirT1 downregulation would result in AMPK inactivation, thereby decreasing sensitivity to cisplatin and doxorubicin, and inducing MDR through SirT1-AMPK signaling.

To conclude, modulating MYC signaling together with anticancer drug treatment targeting MDR-substrate pathways has potential as a novel approach to overcome MDR.

### 3.11 MYC and Intestinal Flora

Gut microbiota is a crucial mediator of numerous physiological processes in humans [386, 387]. Diseases can develop as a result of dysbiosis, which causes dysfunction of the intestinal barrier and alters intercellular/intracellular metabolic pathways and immune responses [386, 388]. It is established that changes in gut microbiota composition are associated with tumorigenesis [388]. Overall, gut microbiota is primarily considered to inhibit cancer occurrence and development resulting from stress-related DNA damage, pro-inflammation, and modulation of the host immune system [389]. Consequently, probiotics and symbiotics are promising strategies to reduce carcinogenic risk via intestinal microbiota modulation [390].

#### 3.11.1 Proliferating Cell Nuclear Antigen (PCNA)

Microbiota bacteria influence MYC expression and regulate its mediation of carcinogenesis-related gene expression, modulating the function of intestinal flora metabolic pathway function, and initiating inflammatory responses to induce programmed cell death [389]. Cruz et al. used the probiotic (PRO) VSL#3, containing eight species of freeze-dried bacteria (*Lactobacillus casei, Lactobacillus plantarum, Lactobacillus acidophilus, Lactobacillus delbrueckii, Bifidobacterium longum, Bifidobacterium breve, Bifidobacterium infantis*, and *Streptococcus salivarius*) from the human gastrointestinal tract, as well as a PRO VSL#3 combined with PBY (a yacon-based product) formed symbiotic (SYN), and assessed their effects on metabolic pathways in the intestinal tract, and inhibition of colorectal carcinogenesis, through the suppression of the c-MYC and PCNA oncogenes [390, 391]. The SYN group showed enhancement of specific metabolic pathways, including biosynthesis of essential components (amino acids, vitamins, and saccharide subunits) needed for microbiota support, whereas the control and PRO groups tended to be enriched for pathways involved in generation of nucleosides and nucleotides [390]. Further, c-MYC and PCNA expression were downregulated in the SYN group relative to the control and PRO groups; there were no significant differences in p53 and caspase-3 levels among the groups [390]. Additionally, cytokine (IL-2, IL-4, TNF, and IFN) levels were higher in the SYN group than those in the PRO and control groups, and elevated cytokine levels trigger anti-inflammatory responses [390]. Hence, the study demonstrated that modulation of intestinal flora confers specific benefits in enhancing microorganism metabolic pathways which promote vitamin production to induce antineoplastic effects on DNA metabolism, apoptosis, and anti-inflammatory activity [390], leading to suppression of MYC and PCNA expression levels and providing enhanced carcinogenesis control.

#### 3.11.2 Protease Lon

The uropathogenic *Escherichia coli* protease, Lon, can reduce c-MYC expression in animal and human models and improve the prognosis of patients with c-MYC-induced cancer [392]. A combination of systematic gene deletion and proteomics experiments demonstrated that bacterial culture supernatants contained a specific protease (Lon) that Lon is a potent MYC inhibitor [392]. Lon protease was purified for recombinant expression and delivered into mouse models of MYC-dependent bladder and colon cancer via peroral or intravenous routes [392]. The results demonstrated that c-MYC degraded rapidly after *in vivo* Lon injection, suggesting that Lon degrades c-MYC, or accelerates endogenous pathways for c-MYC degradation [392]. This finding suggests that Lon offers a promising approach for MYC inhibition to control MYC-dependent carcinogenesis.

#### 3.11.3 Casein Kinase 1 Alpha 1 (CK1α1)

In addition to by-product secretions of intestinal bacteria, chronic bacterial infections can also mediate c-MYC degradation. Pathogenic bacteria alter c-MYC expression through a mechanism involving CK1α1 activation, which is controlled by α-hemolysin (α-hly) [392, 393]. c-MYC Serine-252 is phosphorylated in response to CK1α1 activation, triggering its proteasomal degradation, while α-hly is a pore-forming toxin associated with ABC transporters, that facilitates CK1α1 entry into cells via Ca^2+^ flux, and interacts with c-MYC for degradation [392, 393]. Based on these biochemical interactions, c-MYC was shown to be degraded in infected cells via the effects of CK1α1 activation induced by α-Hly [392].

Overall, microbial flora may contribute to protecting cells against MYC-mediated oncogenic transformation. Further research is needed to identify compounds that can modify intestinal microbiota composition and activity, as well as to develop biomarkers and screen for relevant carcinogens, which will enable more accurate prediction of carcinogenesis based on microbial signatures.

## 4. MYC Modulators as Cancer Treatments

Several MYC modulators/inhibitors, including Food and Drug Administration (FDA) -approved drugs, agents in clinical trials, chemical tools, and active compounds from natural products or herbal medicine, have been used, or are in the clinical trial or pre-clinical investigation phases, for cancer therapy.

### 4.1 MYC Therapy Patent Landscape

Given the role of MYC in tumorigenesis, the design of MYC modulators is particularly important, and several strategies have been proposed in the last decade. Since the mid-1990s, the discovery and design of anticancer drugs based on MYC modulators has increased [394]. MYC modulators have become increasingly important for further research in this field, since patents have been awarded for their discovery, synthesis, and application; however, design and development of direct MYC modulators is highly challenging, because the network of independent pockets in the protein make modulator binding difficult and their half-lives are short. Nevertheless, MYC cannot simply be assumed to be an unreachable target, despite the challenge of finding direct inhibitors or binders [394]. Efficient methods of achieving MYC modulation appear to include interfering with transcription of MYC and its cofactors, blocking their protein-protein interactions, and influencing their associated signaling pathways, and various MYC modulators have been developed based on these features [394].

**Figure 5.**
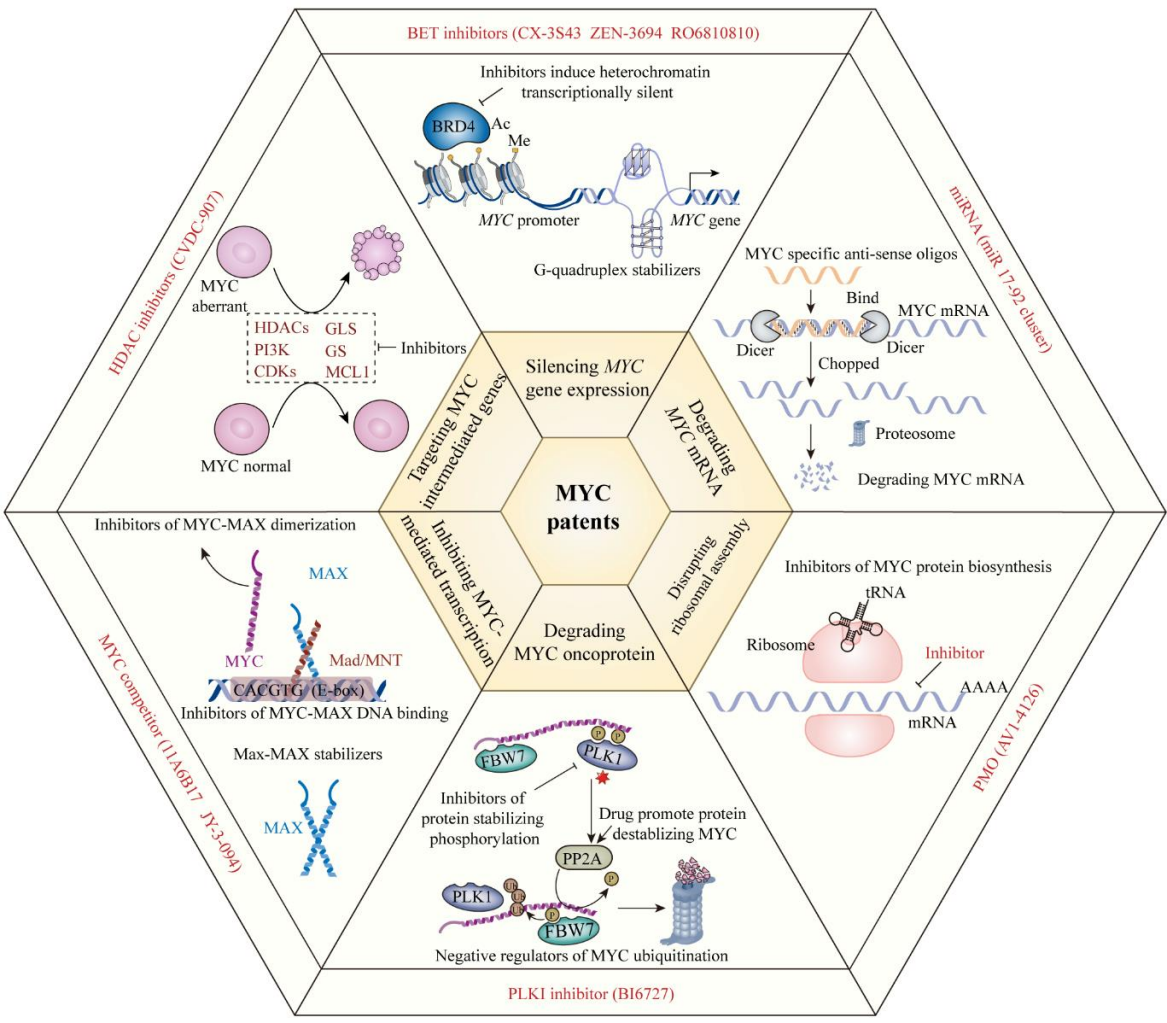
**MYC patent landscapes**. HDACs: Histone deacetylase; PI3K: phosphoinositide 3-kinase; CDKs: Cyclin-dependent kinase; GLS: Glutaminase; GS: Glutamine synthetase; MCL-1: myeloid cell leukemia sequence 1 protein; BRD4: Bromodomain-containing protein 4; Ac; Acetylated; Me: Methylated; FBW7: F-box and WD repeat domain containing 7; PLK1: polo-like kinase 1; PP2A: Protein phosphatase 2.

An overview of several therapeutic MYC modulator patents is provided below (Fig. 5); some of these are FDA-approved and others are still in the clinical trial or preclinical phase.

### 4.2 FDA-approved MYC Drugs

The FDA has published a standard for approving drugs for treatment of MYC-mediated cancers, which includes three drugs, everolimus, sirolimus, and temsirolimus, that exploit vulnerabilities in the MYC-TOR interaction, as well as one medication (mycophenolic acid, IMPDH) that targets MYC directly [395]. Further, various HDAC inhibitors are in development, some of which have already been approved by the FDA [396]. Recently, some agents against additional synthetic lethal targets of MYC have successfully advanced to clinical trials.

### 4.3 Small Molecule Modulators of MYC in the Pre-clinical Stage

MYC inhibition can rapidly reduce tumors, highlighting its importance [397]. Small molecules inhibiting MYC/MAX dimerization, as well as RNA interference (miRNA, siRNA) to downregulate MYC translation, are effective ways of directly preventing MYC activity [397].

OmoMYC agents have recently gained prominence as dominant-negative MYC proteins [398]. Blockage of MYC by OmoMYC initially appeared challenging, due to the anticipated side effects [398]. Nonetheless, tests in animal models suggest that the side effects are relatively mild [399]. OmoMYC inhibits MYC activity by infiltrating cells through spontaneous cell penetration [398]. In addition, OmoMYC provided significant benefit when delivered directly to cells and administered systemically in NSCLC models [399]. OmoMYC inhibits MYC via two mechanisms: (1) interfering with MYC dimerization and (2) binding to E-boxes [400]. Aside from OmoMYC, MYC/MAX destabilizers, such as IIA6B17, 10058-F4, and 10,074-G5, and their derivatives, 3jc48-3, JY-3-094, and 3JC-91-2, can also inhibit MYC/MAX complex formation [401-403]. IIA6B17 has the same leucine zipper structure as c-Jun (another tumorigenesis hallmark) and can exert anti-c-Jun activity [404, 405], and hence has poor selectivity and specificity as a MYC inhibitor [404, 405]. JY-3-094 and 3JC48-3 inhibit MYC/MAX dimerization in cells overexpressing MYC and reduce their proliferation [406, 407].

Some small molecules, such as MYCro1, MYCro2, and MYCro3, can inhibit human cancer cell proliferation in xenografts containing MYC-amplified cells [408, 409]. Further, metastatic HER-2-negative breast tumors become significantly more responsive on treatment with MyCro3 together with Palbociclib and CDK4/6 inhibitors [410]. In addition, Mycro3 had superior pharmacokinetic properties and decreased tumor size more than other c-MYC inhibitors in a KRas-driven pancreatic ductal adenocarcinoma mouse model [403, 411], suggesting that direct inhibition of MYC can improve the effects of other targeted therapies both *in vivo* and *in vitro*.

MYCMI-6 was recently identified as a direct MYC inhibitor, independent of biomacromolecules, which can block MYC-driven transcription, by binding selectively to the MYC bHLHZip domain to prevent MYC dimerization with MAX [412, 413]. MYCMI-6 inhibits proliferation and promotes apoptosis of breast cancer cells [414, 415]. In an assisted proteolysis study, Han et al. found that two MYC inhibitors, MYCi361 and MYCi975, block interaction of MYC with its canonical partner, MAX [416, 417]. Blocking MYC increases its degradation, impairs MYC-mediated gene expression, and suppresses tumor growth [416, 417]. These inhibitors can also phosphorylate the MYC Thr-58 residue, facilitating proteasomal degradation of MYC protein [418].

KI-MS2-008 and NSC13728 provide another approach to reducing MYC protein levels and expression of its target genes by stabilizing the MAX homodimer to induce cell growth arrest and differentiation [419-421]. *In vivo* tests showed that KI-MS2-008 and NSC13728 interfere with tumor cell proliferation and prevent cells growth [419, 422] , which may be useful together with monoclonal antibody treatment against the immune checkpoints, PD-1 or PD-L1. KI-MS2-008 can also synergize with the anti-tumor effects of MAX homodimer stabilizers [423, 424]. Furthermore, the transcription repressors, MXD1 and MAD, also inhibit MYC-mediated gene activation [425, 426] , by coupling with MAX to hijack E-box regions of target genes and inhibit MYC-mediated transcription regulation [425-427]. By contrast, JKY-2-169 binds to the MYC-MAX heterodimer and, instead of disrupting heterodimer complex formation, it perturbs MYC/MAX complex binding to canonical DNA E-boxes, antagonizing cancer cell proliferation, cell cycle arrest, and apoptosis in MYC-driven cells [428, 429].

### 4.4 Small Molecule Modulators of MYC at the Clinical Trial Stage

#### 4.4.1 Direct MYC Inhibition

An alternative method for inhibiting MYC translation is transport of siRNAs into cells; however, robust transporters are required for si/miRNAs to function effectively [397]. During clinical trials of therapy for solid tumors, an EnCore lipid nanoparticle enclosing a MYC-specific si/miRNA was used to regress tumor cell growth [397, 430].

Transfection of siRNA in c-MYC-driven cells upregulates *Let-7a, miR-16, miR-29b*, and *miR-494* expression, which target MYC translation and reduce its expression, inhibiting tumor growth and spread by influencing the cell cycle, and triggering apoptosis pathways, limiting ovarian and pancreatic cancer metastasis [431-433]. MYC translation can also be inhibited using the phosphorodiamidate morpholino oligomer (PMO), AVI-4126 [434, 435]. PMOs perturb ribosomal assembly, thereby prohibiting *MYC* mRNA transcription. Clinical trials assessing the effects of AVI-4126 against c-MYC have been conducted in multiple cancer types and related disease models, with promising results, allowing this PMO to progress to human clinical trials. AVI-4126 was the subject of a phase I clinical study to investigate PMO bioavailability in surgically excised adenocarcinomas of the prostate and breast [435].

#### 4.4.2 Indirect MYC Inhibition

Targeting MYC regulating factors can indirectly inhibit MYC, providing flexibility. Therefore, investigation of indirect MYC inhibitors is desirable and we provide a list of them below, some of which have been approved for clinical trials.

##### 4.4.2.1 BET Family Inhibitors

The BET protein family comprises BRD1, BRD2, BRD3, and BRD4 [436] , and BET inhibitors (iBETs) reduce MYC-related oncoprotein expression levels, decreasing the risk of tumor development [436].

Common iBETs include ZEN-3694 and RO6870810 (formerly TEN-010), which bind to the extra-terminal bromodomain to inhibit the BET pathway. ZEN-3694 is currently under clinical investigation (phase II), while TEN-010 is in phase I clinical trials [437, 438]. A number of targeted therapies appear to exhibit drug resistance due to MYC overexpression. To resolve this issue, combination treatment with ZEN-3694 and Enzalutamide acts synergistically by blocking androgen receptors, leading to better prognosis for patients with PCa (NCT04471974) [439]. TEN-010 is also undergoing clinical trials for use in the treatment of acute myeloid leukemia (AML), myeloid dysplastic syndrome, and solid tumors (NCT02308761, NCT01987362) [438].

The BET inhibitor, BMS-986158, is well-tolerated in treatment of advanced cancers [440] , with only an isolated report of thrombocytopenia as a side effect [441]. BMS-986158 has a longer half-life than other iBETs, as well as an impressive pharmacodynamic profile [442]. Further, an iBET taken orally has also been tested for treatment of NUT carcinoma with molibresib (GSK525762), and preliminary findings from phase I clinical trials have led to recommendation of progression to a phase II trial [443].

The orally bioavailable iBETs, AZD5153 and OTX015, could be used to target BRD2, BRD3, and BRD4 [438, 444]. The bivalent iBET, AZD5153, exhibits additional antitumor activity against cancer xenografts relative to monovalent iBETs [445]. Notably, AZD5153 does not regulate apoptosis factors, such as BCL2 anti-apoptosis family members or BCL3 pro-apoptotic proteins [446] , rather its inhibition occurs by altering the mTOR pathway to modulate MYC, E2F, and HEXIM1 expression levels, thereby inhibiting tumor cell growth and killing tumor cells [444, 445]. A synergistic effect was observed between AZD5153 and the BCL2 inhibitor, AZD4320, in cancer treatment [446]. Further, OTX015 showed significant anti-tumor effects on solid tumors, such as neuroblastoma and mesothelioma, as well as hematological cancers [447-449]. These two drugs (AZD5153 and OTX015) are now entering clinical stage investigation as treatments for various diseases.

The iBET, BI894999, affects MYC and HEXIM1 in AML cells in a similar manner to AZD5153 [450]. Taken together with a CDK9 (cyclin dependent kinase 9) inhibitor, this particular iBET causes an apoptotic response via repression of super-enhancer-associated MYC transcription [446].

##### 4.4.2.2 MCL-1 Inhibitors

MCL-1 can promote MYC-induced myeloid leukemogenesis [451]; hence, MCL-1 inhibitors are a potential therapeutic option for targeting tumorigenesis and drug resistance caused by high MCL-1 levels.

The selective small-molecule, AZD5991, is a promising candidate for treating AML; its ability to induce BAK-dependent apoptosis, as well as its significant antitumor properties, have led to its selection as a treatment option for patients with relapsed or refractory AML in clinical trials [452]. As well as being used alone, AZD5991 has been combined with other agents, such as Bortezomib (which inhibits 26S proteasomes) and venetoclax (which inhibits BCL-2), and the effects examined in carcinoma models [452]. Further, the MCL-1 inhibitor, S64315 (MIK665), induces BAX/BAK-mediated apoptosis, acting in a somewhat similar manner to AZD5991 to inhibit MYC activity [453]. MCL-1 inhibition is potentially a promising approach for cancers involving MYC, due to the cooperative interactions between BCL-2 and MYC [454].

##### 4.4.2.3 BCR-signaling Inhibitors

MYC can be activated by BCR signaling and MYC induction during tumorigenesis can be attributed to BCR-signaling mediators, such as BTK [455, 456].

The BTK inhibitor, ARQ531, can also inhibit SRC kinases and ERK signaling pathways involved in BCR signaling [457]. In a chronic lymphocytic leukemia model, ARQ531 showed strong inhibitory potency against BCR-induced cancer cells by repressing a broad range of BCR-signaling factors, and is currently being tested against MYC-related hematological neoplasms in a phase I clinical trial (NCT03162536), due to its potential to overcome resistance to some existing BCR inhibitors [457].

##### 4.4.2.4 PI3K and HDAC Inhibitors

Given the short half-life of MYC, eukaryotic translation initiation factor 4 (eIF4) plays an important role in MYC translation [458]. Several upstream signals are activated in response to hyperphosphorylation of eIF4E-binding protein 1 (4E-BP1), which sequesters eIF4E [458]. MYC translation can be initiated by PI3K, independent of 4E-BP1 phosphorylation [458]. Further, MYC expression can be stabilized by post-transcriptional modifications mediated by the HDAC family [459]. Fimepinostat (CUDC-907) inhibits *MYC* mRNA translation and stabilization by inhibiting PI3K and HDAC proteins [460].

The efficacy of PI3K inhibitors is impeded by simultaneous activation of other survival-supporting pathways [461]. Hence, double inhibition is required and a dual inhibitor, CUDC-907, has been developed to suppress PI3Kδ and HDAC expression, and appears to overcome the limitations of inhibitors targeting PI3K alone [462]. CUDC-907 has undergone clinical trials in various hematological cancers and shown promising results in terms of tolerability, safety, and efficacy [460, 463, 464].

##### 4.4.2.5. CDK Inhibitors

CDK9 couples with cyclin T1 to form positive transcription elongation factor b (p-TEFb), which phosphorylates a serine residue on the RNA Polymerase II C-terminal repeat domain (CTD) [465, 466]. Binding of MYC and p-TEFb activates RNA polymerase II, enhancing transcriptional activity and this process drives survival in MYC-induced hepatocellular carcinoma models [213, 465, 467].

Dinaciclib is a CDK inhibitor, which suppresses the kinase activities of CDK1, CDK2, cyclin dependent kinase 5 (CDK5), and CDK9 and is currently undergoing phase I/II clinical trials for use against various tumors [468]. The trial results have revealed prominent CDK9 inhibition effects of Dinaciclib, which prevents binding of MYC and p-TEFb [469]. In addition, Dinaciclib also inhibits MCL-1, reducing its expression and inducing apoptosis [469].

A second CDK inhibitor, TG02, inhibits CDK activity via inhibition of the CDK1, CDK2, cyclin dependent kinase 7 (CDK7), CDK9, Janus kinase 2 (JAK2), and fms related receptor tyrosine kinase 3 (FLT3) pathways [470]. A potential benefit of this multi-kinase inhibitor is that it can inhibit the activities of various CDKs, as well as blocking BCR-signaling mediators, contributing to superior antitumor activity than that achieved by complementary MYC inhibition [470, 471]. Examination of the effects of TG02 on hematological malignancies has generated promising safety, pharmacokinetics, and pharmacodynamics data, allowing this drug to move into clinical trials [470, 471].

##### 4.4.2.6 G-quadruplex (G4) Stabilizers

Eighty to ninety percent of *MYC* gene transcription is regulated by nuclease hypersensitivity element III1 (NHE III1). A G4 is created at a specific site in NHE III1, acting as a silencer [472], and drugs targeting this specific region can stabilize G4 structures, which generally promotes apoptosis [473]. Compounds such as CX-3543 can stabilize the *MYC* promoter by selective interaction with the G4 site, interfering with formation of nucleolin/rDNA G4, and inducing apoptosis [474]. Hence, CX-3543 has MYC modulatory properties and is the first G4 stabilizer to undergo clinical trials [475].

### 4.5 Herbal Medicine Modulators of MYC for Cancer Treatment

**Figure 6.**
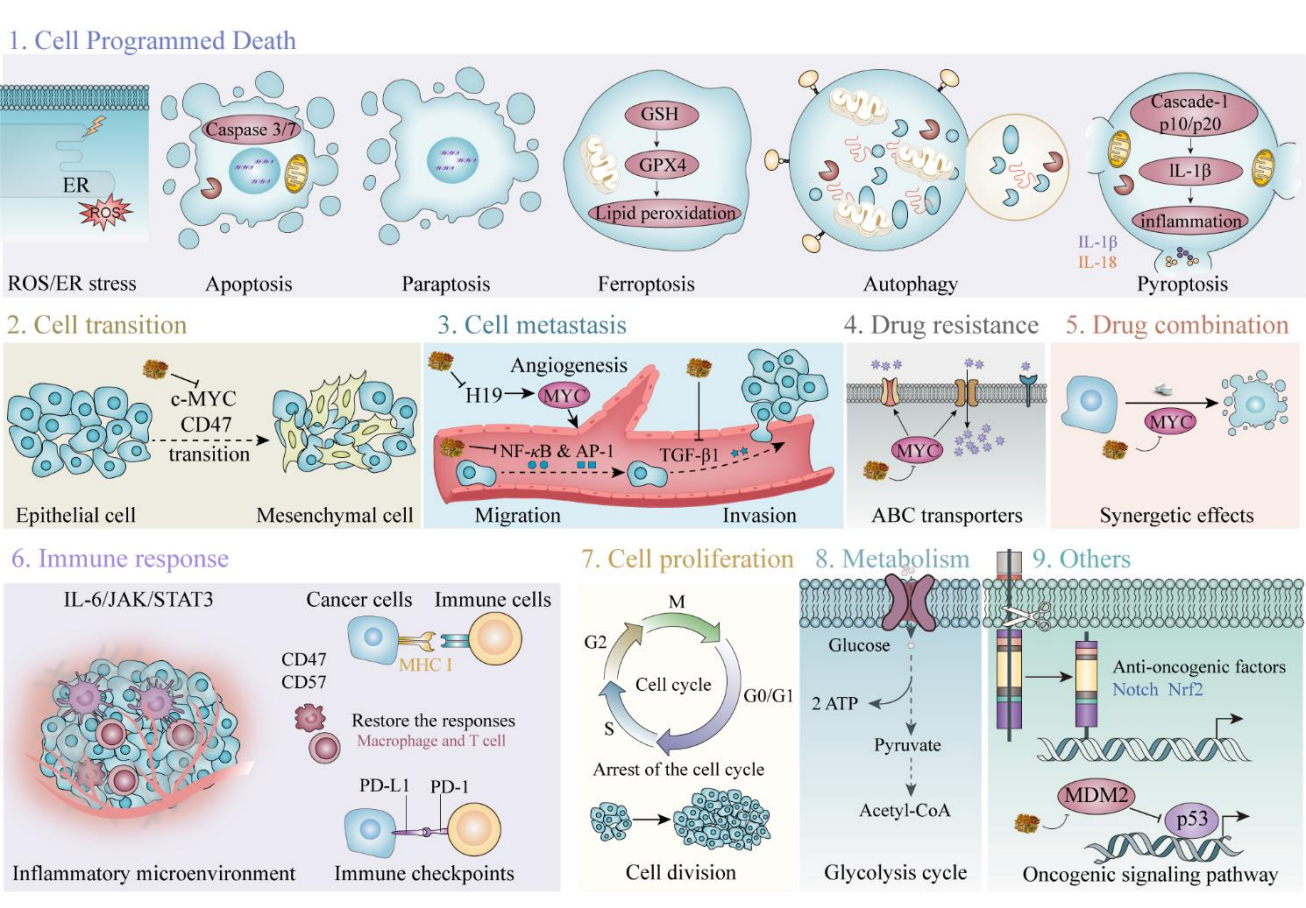
**The roles of herbal medicine act in modulating MYC-driven malignant cells**. ABC Transporter: ATP-binding cassette transporters; Acetyl-CoA: acetyl coenzyme A; AP-1: Activator protein 1; CD 47: Cluster of Differentiation 47; CD57: Cluster of Differentiation 57; ER: Endoplasmic Reticulum; G0: gap phase; G1 phase: gap 1 phase; G2: Growth 2 phase; GPX4: Glutathione peroxidase 4; GSH: glutathione; H19: H19 Imprinted Maternally Expressed Transcript; IL-1β: Interleukin-1 Beta; IL-18: Interleukin-18; IL-6: Interleukin-6; JAK: Janus kinase; M: Mitosis phase; MDM2: murine double minute 2; NF-κB: Nuclear factor kappa B; Notch: Neurogenic locus notch homolog protein; Nrf2: nuclear factor erythroid 2-related factor 2; PD-1: Programmed cell death protein 1; PD-L1: Programmed death ligand-1; S: Synthesis Phase; STAT3: Signal Transducer And Activator Of Transcription 3; TGF-β1: Transforming growth factor beta-1.

Due to the great contribution of artemisinin for treating malaria, Prof. Tu Youyou won the Nobel Prize in Physiology or Medicine in 2015. Along with the modernization of traditional medicine, herbal medicines have become accepted alternative treatments for human diseases, including cancer, coronavirus, and inflammatory conditions, among others, due to their significant benefits of cost-effectiveness and promising medical safety profiles [476-481]. Studies of phytochemicals derived from medicinal herbs have shown significant inhibition of MYC-dependent cancer cell growth via different molecular mechanisms [482-486] (Fig. 6).

**Figure 7.**
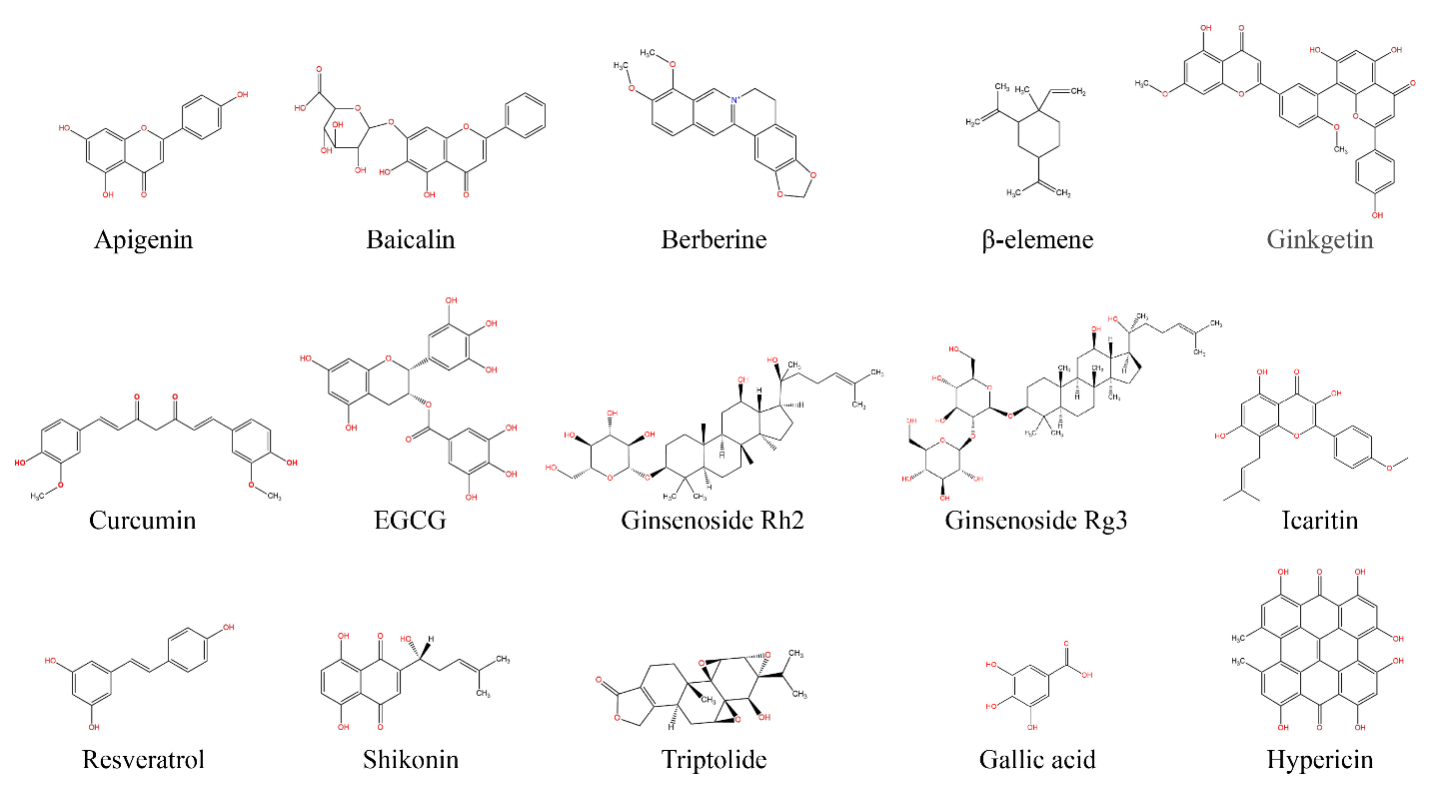
**The chemical structure of natural products extracted from herbal medicine**. Apigenin, baicalin, berberine, β-elemene, ginkgetin, curcumin, EGCG ((-)-Epigallocatechin-3-gallate), ginsenoside Rh2, ginsenoside Rg3, icaritin, resveratrol, shikonin, triptolide, gallic acid, and hypericin.

#### 4.5.1 Apigenin

Apigenin (4′,5,7-trihydroxyflavone) (Fig. 7), which is extracted from *Apium graveolens* L., exhibits potential effects against multiple MYC-driven cancers, and has vital roles in numerous bio-modulatory activities [487]. Apigenin enhances c-MYC protein expression in a dose- and time-dependent manner, along with phosphorylation of p38 and p53, in anaplastic thyroid carcinoma cells; however, apigenin administration suppressed c-MYC activity in other tumor cells [487]. A combination of apigenin and N-MYC knockdown led to better outcomes in suppressing malignant neuroblastoma [488]. Furthermore, apigenin can inhibit the upstream Wnt/β-catenin axis via initiation of the autophagy-lysosomal pathway, a sophisticated signaling network involving c-MYC [489]. Abundant cytokine-associated genes are also disrupted by apigenin, which targets glycogen synthase kinase-3β (GSK-3β), contributing to cancer immunity regulation through a feedback loop between cancer cells and the inflammation-related microenvironment [490, 491]. Interestingly, cancer cells treated with apigenin exhibited fewer ROS-triggered events, contrary to the paradigm of ROS-induced apoptosis, demonstrating that parallel pathways are regulated by p53 and STAT3 in apigenin-mediated anti-tumor events [492]. Moreover, apigenin regulates glycolysis in CRC by targeting PKM2 [493]. Hence, systemic cancer inhibition by apigenin is established via multiple layers of regulatory systems involving cell death, cell metabolism, cancer metastasis, cellular redox balance, and even telomerase remodeling [494].

#### 4.5.2 Artemisinin

The anti-malaria agent artemisinin, which is extracted from *Artemisia annua* L., also has potential for application in cancer management. The Wnt/β-catenin signaling pathway is suppressed in both esophageal cancer and clear cell renal cell carcinoma [495]. c-MYC has indispensable roles in artemisinin-induced anti-cancer networks, as both its mRNA and protein levels are inhibited by artemisinin. Artemisinin can directly induce cancer cell apoptosis, partially attributable to inhibition of the AKT pathway, and promotes the survival of tumor xenograft mice [496]. Interestingly, artemisinin can improve T cell-triggered immune responses, but has limited toxic side effects in other organs [497]. Metabolic remodeling and ferroptosis regulate artemisinin activities, but the relationship between these processes and the role of MYC requires further evaluation [498, 499].

#### 4.5.3 Baicalin

Baicalin, and its deglycosylated derivative, baicalein, regulate multiple cancers, and are major compounds derived from *Scutellaria baicalensis* Georgi [500]. Accumulated molecular biology and system pharmacology studies have revealed a complex signaling network, comprising numerous bio-modulators and their interactions, which is induced by baicalin [500, 501]. Furthermore, investigation of the dose-dependent anti-cancer effects of baicalin has revealed various functional markers with different sensitivities to this compound [500]. In general, pro-oncogenic modulators, such as VEGF, NF-κB, and c-MYC, are particularly inhibited by baicalin at both the transcription and translation levels [502]. Meanwhile, Notch, PI3K/AKT, and MAPK signaling mediate the effects of baicalin in restraining malignancies [503]. Additionally, researchers have used various approaches, based on systematic biological analyses of intersected targets of baicalin and diseases, to determine the molecular relationships underlying the effects of baicalin treatment [504]. c-MYC-driven onco-miRNAs and competitive endogenous RNAs have been captured in experiments to identify molecules that contribute to the pro-apoptotic effects of baicalin in cancer [505]. Baicalin also has multiple effects on chromosomal rearrangement, immune checkpoint monitoring, and glucose metabolism [506]; however, understanding of the anti-cancer functions of baicalin, especially in MYC regulation, remains limited, which is impeding drug development.

#### 4.5.4 Berberine

Berberine is an isoquinoline alkaloid, mostly derived from *Coptis chinensis* Franch., and has anti-cancer efficacy which has been evaluated in pre-clinical and clinical studies [507]. Berberine influences various bio-systems to holistically restrain tumorigenesis. Berberine directly binds to retinoid X receptor α (RXRα), resulting in β-catenin degradation and concomitant c-MYC inhibition [508]. Further, berberine can stabilize c-MYC G4 structures, indicating parallel targets or associated signaling pathways indirectly impacted by berberine during anti-cancer treatment [509]. Glucose and glutamine metabolism triggered by PI3K/AKT can be beneficially modulated by berberine [510]. Berberine can also shift the TME, comprising cancer stem cells, immune cells, and cytokines, toward a more pro-apoptotic milieu [511]. Among the multiple layers of regulation by berberine, differentiation 47 (CD47) suppression initiated by c-MYC, can enhance macrophage phagocytosis to treat diffuse large B-cell lymphoma [512]. Interestingly, reversal of drug resistance induced by berberine treatment relies on c-MYC-associated signaling axes, such as Nrf2 and STAT3, supporting the potential modulation of MYC by berberine [513]. Further analyses will involve investigation of cell death, including through ferroptosis and pyroptosis, potentially induced by berberine, to determine how berberine influences multiple bio-systems.

#### 4.5.5 β-elemene

β-elemene is a bioactive natural product extracted from *Rhizoma zedoariae* oil, with manifold anti-cancer effects [514]. A network pharmacology study discovered dozens of potential β-elemene targets, and eventually focused on chromosome 3 open reading frame 21 (C3orf21) for its tumor suppression capacity [514]. The inhibitory effects of β-elemene may partially depend on C3orf21, as C3orf21 silencing rescued the suppression of carcinogenesis modulators, such as c-MYC and cyclin D1 [514]. Meanwhile, cancer cell apoptosis induced by β-elemene is triggered by the Wnt/β-catenin signaling pathway, which engages in molecular crosstalk with the TCF7/Sox2 axis [515], allowing construction of a sophisticated β-elemene-regulated signaling network involved in attenuation of cancer progression, including EMT and MDR [516]. More effort is required to identify precise targets bound by β-elemene and develop a comprehensive understanding of the mechanisms of action of this compound.

#### 4.5.6 Curcumin

The polyphenol, curcumin, is the main active ingredient in turmeric, a spice widely recognized for its medicinal properties including anti-inflammatory and anti-oxidative activities [517, 518]. Curcumin can reduce MYC expression, followed by histone 19 (H19) induction, leading to regulation of specific pro-proliferative TFs in the pro-metastasis microenvironment [519-522]. Furthermore, curcumin can reverse these processes by increasing levels of tumor suppressors, such as p53, and inducing apoptosis in an EZH2-*miR-101* reciprocal negative feedback loop [519, 523, 524]. Hence, curcumin has potential as an anti-cancer drug to suppress MYC-dependent tumor proliferation and warrants further clinical trials.

#### 4.5.7 (-)-Epigallocatechin-3-gallate (EGCG)

EGCG is a biological polyphenol commonly detected in green tea [525]. Numerous studies have investigated the anti-cancer potential of EGCG and its benefits, such as reversal of drug resistance and inhibition of cancer stem cells [525]. Proteomics analysis demonstrated that the DEAD-box RNA helicase, p68, is a binding target of EGCG [526]. Other molecules predicted to be involved in EGCG-induced apoptosis and autophagy are enriched in the mitochondria-associated redox biosystem [527]. The Wnt/β-catenin signaling pathway is suppressed by EGCG in a c-MYC-dependent manner in different tumor types [528]. Other canonical modulators that interfere with cancer growth, including Akt, ERK1/2, and NF-κB, are also inhibited by EGCG to some extent [529]; however, the precise relationships between EGCG and c-MYC require further investigation, as few studies have focused on whether EGCG directly influences c-MYC expression and activation.

#### 4.5.8 Gallic acid

Gallic acid is a phenolic compound commonly found in *Rheum palmatum* L., *Cornus officinalis* Sieb. et Zucc., and tea. and its anticancer properties have been demonstrated *in vivo* and *in vitro* [530, 531]. Gallic acid can induce apoptosis and inhibit metastasis through the Ras/ERK pathway with downregulated c-MYC protein expression [531]. Gallic acid is usually used together with antitumor agents, such as temozolomide, paclitaxel and carboplatin, and has potential to reverse drug resistance, in which MYC may participate [532, 533]. There have been few studies of the anti-cancer activity of gallic acid particularly its role in regulating MYC, which limits precise understanding of its effects in this context.

#### 4.5.9 Ginkgetin

Ginkgetin is a naturally occurring bioflavonoid originating from *Ginkgo biloba* leaves, and is effective in treating NSCLC via initiation of autophagy [534]. Ginkgetin is predicted to exhibit preferential binding affinity for the c-MYC G4 and to stabilize and repress c-MYC transcriptional activity, thereby inhibiting MYC-driven myeloma growth. Experimental results were consistent with this prediction, since both c-MYC transcript and protein were downregulated by ginkgetin [535]. Combination of the autophagic characteristics of ginkgetin with addition of cisplatin to induce ferroptosis, could trigger increased anti-cancer effects of these non-apoptotic programmed cell death pathways [534]. Hence, there may be future opportunities to develop more potent direct inhibitors of c-MYC by targeting the MYC G4 region with ginkgetin.

#### 4.5.10 Ginsenosides

Ginsenosides are derived from *Panax ginseng* C.A.Mey. and *Panax notoginseng* (Burk.) F. H. Chen. Various ginsenoside homologs exert anti-cancer effects that are dependent on host responses [536]. Ginsenoside Rh2 targets Annexin A2 and can induce apoptosis and paraptosis by suppressing the TFs, NF-κB and AP-1 [537]. Consequently, downstream pathways involving c-MYC are inhibited and the pro-oncogenic functions of tumor glycolysis are restrained [538]. These events are also relevant to Ginsenoside Rg3 treatment, which disrupts the biological functions of ZFP91 [539]. The ginsenoside Rk1 alters c-MYC, which engages in cross-talk with ERK during glutamine metabolism, and exerts anti-cancer activity with lower cytotoxicity than sorafenib [540]. Other ginsenosides, including Rg1, Rh4, and Rg5, with specific structures and functions, can inhibit cancers accompanied by suppression of c-MYC, indicating that bio-modulators induced by these natural homologs have common features [541].

#### 4.5.11 Hypericin

Hypericin is an active natural polycyclic quinone component extracted from most plants of the genus *Hypericum* and has anti-tumor effects in the MCF-7 breast cancer cell model [542, 543]. The antiproliferative or cytotoxic effects of hypericin have been demonstrated in numerous studies [542-546]. Among its anticancer effects, hypericin can inhibit various genes associated with Bcl-2, MYC, and MDm2, to affect expression levels of these oncoproteins. Hypericin also targets the heat shock protein 90 (HSP90) chaperone to degrade Plk, CDK4, and Raf1 proteins. Further, hypericin upregulates expression of the *p53, p21*, and *Bax* genes, leading to caspase activation, cytokine release, cell cycle arrest at metaphase, and promotion of apoptosis [542, 547].

#### 4.5.12 Icaritin

Icaritin, a hydrolysis product of icariin extracted from the *Epimedium* genus, is the first small molecule immunomodulator approved by National Medical Products Administration of China in 2022, and used for hepatocellular carcinoma treatment [548]. c-MYC protein expression is inhibited by icaritin in Burkitt lymphoma and AML cells [549, 550], triggering activation of caspase-9 and PARP, as well as eventually leading to cell cycle arrest (S phase) and apoptosis/necrosis promotion [550, 551]. Furthermore, MAPK/ERK/JNK and PI3K/AKT signaling are regulated by icaritin to inhibit phosphorylation of ERK and Akt, which induces apoptosis to reduce myeloma cell growth in AML [550, 552, 553]. In addition, icaritin can target MyD88 and IkBα to inhibit IL-6/JAK/STAT3 signaling, thereby regulating the TME and inhibiting tumor cell growth [554-556]. Further, a study reported re-sensitization of cancer cells to medicinal agents by icaritin, as well as the attenuation of side effects indicating the potential for clinical co-treatment including icaritin as a supplementary drug [557]. Further evaluation of icaritin for application in regulation of hematopoiesis and hematological cancer therapy is warranted, owing to its apoptotic properties and immunomodulatory effects.

#### 4.5.13 Polysaccharides

As important biomacromolecules in herbal medicines, polysaccharides exhibit effective antitumor activity by inducing apoptosis, suppressing tumor metastasis, arresting the cell cycle, and promoting immune responses across a wide range of cancers, including lung cancer, gastric cancer, CRC, hepatocellular carcinoma, and renal cell carcinoma [558]. Expression of MYC genes and proteins is implicated in the antitumor mechanisms of polysaccharides derived from herbal medicines. In a lung cancer cell culture model, polysaccharides were shown to have sophisticated roles in regulating MYC and other molecules. Polysaccharides from *Ganoderma lucidum* and *Laminaria japonica* suppressed c-MYC protein levels in a tumor-bearing mouse model of lung cancer, through inactivating ERK and *β*-catenin/TCF4 signaling, respectively [559-561]. Furthermore, polysaccharides from *Ulva prolifera* O.F. Müller contributed to suppression of H_2_O_2_-induced cell invasion by inhibiting MYC-mediated *MMP-9* gene transcription via MAPK signaling in A549 and NCI-H1650 lung cancer cells [562]. In a gastric cancer cell model, polysaccharides extracted from different parts of *Dendrobium huoshanense* downregulated *c-Myc* gene expression in MFC cells and promoted *p53* transcription, thereby enhancing p53-dependent apoptosis [563]. *Albuca bracteata* polysaccharides exhibit anti-colitis-associated-CRC properties by attenuating oxidative stress, regulating inflammation, and increasing the relative abundance of beneficial bacteria in a mouse model, followed by suppression of *c-Myc* gene expression [564, 565]. Combination treatment with *Albuca bracteata* polysaccharides and 5-FU showed synergistic anti-cancer effects in modulating β-catenin signaling and intestinal flora, as well as downregulating c-MYC protein levels in CRC more strongly than 5-FU treatment alone [565]. *Acanthopanax senticosus* polysaccharides could induce apoptosis and cell cycle arrest in G0/G1 phase in HepG2 hepatocellular cells, as well as decreasing c-MYC protein and inhibiting Wnt/β-catenin signaling [566]. Combination of IFN-α2b and polysaccharides from *Lycium barbarum* showed synergistic anti-renal cell carcinoma activity both *in vitro* and *in vivo* [567]. This combination treatment could induce cell death and reduce myeloid-derived suppressor cells by downregulating c-MYC protein [567]. MYC is regarded as a promising target for cancer treatment, and research on herbal medicines with anticancer properties related to MYC regulation has gradually matured, but few studies have focused on the potential anti-cancer effects of polysaccharides from herbal medicines involved in MYC signaling.

#### 4.5.14 Resveratrol

The phenolic compound, resveratrol, belongs to the stilbenoids family, which is characterized by the presence of two linked phenol rings connected by an ethylene bridge, and exhibits strong antitumor activity against various types of cancer [568]. Over 70 species of plants contain resveratrol, particularly grape skin and seeds, and traces of this substance have also been found in red wine and several foods [568]. c-MYC and cyclin-D1 expression are downregulated by resveratrol in human breast cancer cells [569]. Downregulation of c-MYC reduces *miR-17* activity, which alters the expression levels of HLA-A and -B MHC class I proteins [570]. This increases opportunities for immune cell recognition of cancerous peptides or other factors, to initiate elimination of cancer cells via immune responses [569, 570]. Thus, resveratrol is a promising therapeutic drug against certain breast cancers induced by MYC expression. Further investment will be required to progress clinical trials.

#### 4.5.15 Shikonin

Shikonin, a natural product with a naphthoquinone core extracted from Arnebiae Radix, exerts multiple anti-cancer effects, particularly against lymphoma. A study comparing the effects of shikonin and its derivatives in killing leukemia cells identified direct binding of shikonin to c-MYC [571]. The apoptosis triggered by shikonin is attributed to a set of suppressed molecules involved in cross-talk between the MAPK and AKT pathways, in which the MST1-YAP1-TEAD1 axis has parallel impacts [572]. Combined treatments including chemotherapy and shikonin induce synergistic effects and ameliorate MDR in different types of cancer [573]. Interestingly, shikonin suppressed proliferation of MCF-7 cells with high Erα expression; degradation of ERα and suppression of ERα-associated molecules suggested that shikonin may enhance antihormone therapies to control breast cancer [574].

#### 4.5.16 Triptolide

Triptolide, a diterpenoid triepoxide from *Tripterygium wilfordii*, functions in tumor suppression processes, making investigation of its application attractive. Triptolide can induce both apoptosis and pyroptosis, mainly triggered by cell cycle regulators and gasdermin E (GSDME), respectively [575]. Treatment with triptolide inhibits core pro-oncogenic modulators, including c-MYC and CDKs, thereby inducing complex modulation of tumor growth and MDR [576]. Interestingly, the epigenetic alterations caused by triptolide, which inhibit DNA methyltransferase (DNMT)-1 and DNMT-4, are associated with the Wnt/β-catenin signaling pathway [577]. c-MYC can act as a marker to evaluate the effects of triptolide on different bio-functions in carcinoma. Meanwhile, a systemic network, comprising altered mRNA and DNA signatures, has been established to assess the precise molecular mechanisms of action of triptolide, and particularly to explore its interactions with MYC [578].

**Table 1:** Potential MYC modulators from natural products of herbal medicine for cancer treatment.

Compound	Cancer phenotypes	Experimental models	Modulators involved in cancer treatments	Pharmacological effects	References
**Apigenin**	Anaplastic thyroid carcinoma, Malignant neuroblastoma, Colorectal cancer, prostate carcinoma, Pancreatic cancer, Primary effusion lymphoma, Non-small cell lung cancer	*In vitro*: FRO, SK-N-DZ, SK-N-BE2, P19, HCT-116, BxPC-3, PANC-1, PEL, H1975; *In vitro* doses: 0.75-100 μM; *In vivo*: Transgenic adenocarcinoma of the mouse prostate; *In vivo* doses: 20-50 μg/d;	Inhibiting: p-ERK1/2, p-JNK, p21, p27, Bcl2, c-Myc, VEGF, MMP-2, MMP-9, Axin2, cyclin D1, cyclin B1, β-catenin, p-Akt, p-p70, p-4E-BP-1, XIAP, K-Ras, p65, GSK-3β, STAT3, FLIP, Glut1, PKM2, PTBP1, hTERT, HIF-1α Enhancing: p38, p-p53, Bid, Fas, cleaved PARP-1, cleaved caspase-3, SBDP, ICAD, ratio of LC3B II/I, E-cadherin, cytochrome C	Inducing caspase-dependent apoptosis, suppressing cancer cell migration, arresting cell cycle, blocking glycolysis of cancer cells	[487-490, 492-494]
**Artemisinin**	Esophageal cancer, Breast cancer, Clear cell renal cell carcinoma	*In vitro*: EC109, MDA-MB-231, UMRC-2, CAKI-2, 4T1; *In vitro* doses: 1-100 μM; *In vivo*: UMRC-2 tumor-bearing mice; 4T1 tumor-bearing mice; *In vivo* doses: 20-100 mg/kg	Inhibiting: β-catenin, c-Myc, Bcl-2, cyclin D1, PCNA, N-cadherin, Vimentin, Snail, p-Akt, TGF-β Enhancing: Bax, cleaved-caspase 3, cleaved-caspase 9, p-p38, E-cadherin, T-bet, IFN-γ, TNF-α	Inducing caspase-dependent apoptosis, suppressing cancer cell migration and metastasis, promoting T cell-mediated anti-tumor immune responses	[495-499]
**Baicalin**	Ovarian cancer, Jurkat T cell acute lymphoblastic leukemia, Burkitt lymphoma, Erythroleukemia, Colon cancer, Triple negative breast cancer, Osteosarcoma, Hepatocellular carcinoma, Melanoma, B-acute lymphoblastic leukemia	*In vitro*: OVCAR-3, CP70, IOSE364, HL-60, K562, CA46, HCT116, SW480, MDA-MB-231, HT-29; MG63, SMMC-7721, HepG2, SK-MEL-2, A375; *In vitro* doses: 1.25-150 μM *In vivo*: HCT116 tumor-bearing mice, HT-29 tumor-bearing mice, H22 tumor-bearing mice, B16F0 tumor-bearing mice; *In vivo* doses: 50-100 mg/kg	Inhibition: VEGF, HIF-1α, c-Myc, NF-κB, hTERT, Bcl-2, p-Akt, p-IκB, p-Rb, MMP-7, PD-L1, p-STAT3, Glut1, Glut3, p-mTOR, p-p70S6K, p-4E-BP1 Enhancing: cleaved caspase-3, cleaved caspase-8, cleaved caspase-9, Bax, Fas, FasL, Notch1, DEPP, p-Raf1, p-ERK, p16, p27, p-ERK, E-cadherin	Arresting cell cycle progression, inducing apoptosis, suppressing erythroid differentiation, inhibiting cancer cell proliferation, migration, and invasion, enhancing host T cell responses, inducing cell senescence, inhibiting glycolysis	[500-506]
**Berberine**	Colorectal cancer, Liver Cancer, Triple-negative breast cancer, Neuroblastoma, Diffuse large B-cell lymphoma, Non-small cell lung cancer	*In vitro*: KM12C, HCT116, Hep3B, BEL-7404, MDA-MB-231, MCF-7, N2a, LY1, LY8, U2932, H157, H460, H1975, H1975, H460; In *vitro* doses: 12.5-150 μM *In vivo*: KM12C tumor-bearing mice, CT26 tumor-bearing mice, Hep3B tumor-bearing mice, A20 tumor-bearing mice, Lewis-tumor-bearing mice; *In vivo* doses: 4-20 mg/kg	Inhibition: PCNA, β-catenin, Cdc2, c-Myc, RXRα, HIF-1α, SLC1A5, GLS, PSPH, CDK6, DNMT1, CD133, β-Catenin, Sox2, Notch2, Nestin, cyclin D1, cyclin E, Cdk2, Cdk4, Bcl2, Bcl-xl, MMP-2, MMP-9, Vimentin, p-PI3K, p-Akt, Ras-1, Raf-1, p-ERK1/2, CD47, PD-L1, p-STAT3 Enhancing: p-Akt, PIK3CA, MAP2, p21, p27, p53, Bax, Smad, Nectin, Laminin, NCAM, HSP70, E-cadherin	Inducing cancer cell apoptosis, regulating cancer cell metabolism, inhibiting cancer cell proliferation, suppressing glutamine uptake, restoring macrophage and T cell functions in tumor microenvironment	[507-513]
**β-elemene**	Non-small cell lung cancer, Cervical cancer	*In vitro:* A549, PC-9, SiHa, HeLa; *In vitro* doses: 100 ng/ml -70 μg/ml,	Inhibiting: MMP-2, MMP-9, VEGF, cyclin D1, c-Myc, COX-2, Notch1, Bcl-2, β-catenin, TCF7, SOX2, Vimentin Enhancing: PTEN, p15, p53, Bax, E-cadherin	Inducing cancer cell cycle arrest, inhibiting cancer cell proliferation, inhibiting cancer EMT	[514-516]
**Curcumin**	Gastric cancer, Pancreatic cancer, Triple negative breast cancer	*In vitro*: SGC7901, SW1990, MDA-MB-468; *In vitro* doses: 5-50 μM, *In vivo*: SW1990 tumor-bearing mice; In *vivo* doses: 25 mg/kg	Inhibiting: c-Myc, H19, Bcl-2, N-cadherin, E2F-1 Enhancing: p53, Bax, cleaved caspase-3	Inducing cancer cell apoptosis, arresting cancer cell cycle	[518, 520, 522, 524]
**(-)-Epigallocatechin-3-gallate**	Neuroblastoma, Colorectal cancer, Gastric cancer, Oral squamous cell carcinoma, Hepatoblastoma, Breast cancer, Skin cancer	*In vitro*: BE(2)-C, HCT116, SW480, AZ521, SSC-4, HepT1, HepT3, HUH6, HepG2, MDA-MB-231, A431, SCC13; *In vitro* doses: 1-100 μM In *vivo*: HCT116 tumor-bearing mice	Inhibiting: EGFR, MMP-2, MMP-9, COX-2, Notch1, c-Myc, Bmi1, EZH2, p68, β-catenin, cyclin D1, cyclin D2, CDK2, CDK4, p-Tyr Enhancing: PXRγ, cleaved-PARP, Fas, SFRP1, HBP1, CK1α	Limiting tumor cell sphere formation, inducing apoptosis, arresting tumor cell cycle	[525-529]
**Gallic acid**	Breast cancer, Glioblastoma	*In vitro*: MCF-7, U87MG. *In vitro* doses: 300-6000 μM;	Inhibiting: p-Akt, p-JNK, Enhancing: p53, Bax, p-p38, cleaved-casp-3	Inducing apoptosis, re-sensitizing cancer cells to anti-cancer agents	[530-533]
**Ginkgetin**	Non-small-cell lung cancer	*In vitro*: A549; In *vitro* doses:5 μM; *In* vivo: A549 tumor-bearing mice, *In* vivo doses:30 mg/kg	Inhibiting: GPX4, SCL7A1, GSH, HO-1, Nrf2 Enhancing: transferrin, glutamate, cystine, ROS, cleaved caspase-3, cleaved caspase-7, cleaved caspase-9, cleaved PARP	Inducing apoptosis, inducing ferroptosis	[534, 535]
**Ginsenoside Rh2**	Non-small cell lung cancer, Liver carcinoma, Leukemia	*In vitro*: A549, H460, HepG2, NCI-H1975, NCI-H1975/OSIR, HCC827, KG-1a; *In vitro* doses: 10-80 μM	Inhibiting: Bcl-2, ZEB1, N-cadherin, Vimentin, Glut1, PKM2, LDHA, p-STAT3, c-MYC, MMP-3, TCF4, cyclin D1, p65, p50, Annexin A2 Enhancing: Bax, cleaved caspase-3, E-cadherin, HDAC4, cleaved PARP	Inducing apoptosis and paraptosis, inhibiting tumor cell proliferation and invasion, suppressing tumor glycolysis	[536-541, 579-581]
**Ginsenoside Rg3**	Non-small cell lung cancer, Pancreatic ductal adenocarcinoma; Osteosarcoma, Colon cancer	*In vitro*: A549, H1299, PANC-1, BxPC-3, 143B, MG63, SW480; *In vitro* doses: 25-200 μM *In vivo*: Lewis lung carcinoma cells tumor-bearing mice, PANC-1 tumor-bearing mice; *In vivo* doses: 10-30 mg/kg	Inhibiting: NF-κB, MMP-9, ZFP91, MMP-2, MMP-7, N-cadherin, Vimentin, ZEB1, Snail, Twist, β-catenin, c-MYC, cyclin D1, COX-2	Inducing apoptosis, suppressing cancer cell migration	[536-539, 582, 583]
**Hypericin**	Breast cancer	*In vitro:* A2780, HL-60, cBCRP, MDA-MB-175-VII, DA3, SQ2, *In vitro* doses: *0.5-40* μg/ml	Inhibiting: Bcl-2, Raf-1, Plk, cyclin A, cyclin B1, cyclin H, p27 Enhancing: MRP1, p53,	Inducing apoptosis	[542-547]
**Icaritin**	Burkitt lymphoma, Colon cancer, Acute myeloid leukemia, Hepatocellular Carcinoma	*In vitro:* P3HR-1*;* Raji, COLO-205, NB4, HL 60, U937, *In vitro* doses: 1-20 μM; *In* vivo: Hepa1-6 tumor-bearing mice; *In* vivo doses: 70 mg/kg	Inhibiting: Bcl-2, c-Myc, PD-L1, p-IκBα, p-IKKα/β, Bcl-2, cyclin D1, cyclin E, p-ERK, p-AKT Enhancing: cleaved caspase-3, cleaved caspase-7, cleaved caspase-8, cleaved caspase-9, cleaved-PARP, Bax, CD3^+^ T cells, CD8^+^ T cells, IFN-γ, ROS	Inducing adaptive immune responses, inducing apoptosis, attenuating inflammatory microenvironment	[533, 548-550, 552-557]
**Polysaccharides of *Acanthopanax senticosus***	Liver cancer	*In vitro:* HepG2, *In vitro* doses:10-80 mg/L	Inhibiting: c-Myc, Cyclin D1, β-catenin	Inducing cancer cell apoptosis, arresting cancer cell cycle	[566]
**Polysaccharides of *Albuca bracteata***	Colon cancer	*In vitro:* CT26*,* In *vitro doses:*0.05-0.2 mg/ml *In vivo:* AOM/DSS-induced CAC mice, CT26 tumor-bearing mice*; In vivo doses:* 0.5-1 mg/ml	Inhibiting: IFN-γ, IL-6, TNF-α, MDA, p-STAT3, COX-2, Cyclin D1, c-Myc, β-catenin, PCNA, Vimentin, Enhancing: IL-10, GSH, E-cadherin	Attenuating inflammatory microenvironment, rebalancing microbiota proportions, suppressing cancer cell proliferation	[564, 565]
**Polysaccharides of *Dendrobium huoshanense***	Gastric cancer	*In vitro: MFC, In vitro doses:0.025-2.5 mg/ml*	Inhibiting: c-Myc Enhancing: p53	Inducing cancer cell apoptosis	[563]
**Polysaccharides of *Ganoderma lucidum* (Ganoderan)**	Non-small cell lung cancer	*In vitro WI-38, H510A, A549: In vitro* doses:0.25-5 mg/ml *In vivo:* A549 tumor-bearing mice, *In vivo* doses: 10-30 mg/kg	Inhibiting: Ki67, PCNA, N-cadherin, Vimentin, Snail, Bcl-2, Ras, p-MEK1/2, p-ERK1/2, c-Myc Enhancing: E-cadherin, Bax, cleaved caspase-3, cleaved PARP	Inducing cancer cell apoptosis, suppressing cancer cell proliferation, Inhibiting EMT	[559-561]
**Polysaccharides of *Laminaria Japonica***	Non-small cell lung cancer, Liver cancer	*In vitro:* A549, NCI-H292, H22*; In vitro* doses: 5-20 mg/ml *In vivo: A549* tumor-bearing mice *In vivo* doses*:* 5-20 mg/kg	Inhibiting: Ki67, PCNA, Bcl-2, VEGF, N-cadherin, β-catenin, TCF4, c-Myc Enhancing: Bax, cleaved-caspase-3, cleaved caspase-9, E-cadherin	Inducing cancer cell apoptosis, suppressing cancer cell proliferation, inhibiting EMT	[559-561]
**Polysaccharides of *Lycium barbarum***	Renal cell carcinoma	*In vitro*: Renca*, In vitro* doses: 200 μg/ml *In vivo:* Renca tumor-bearing mice, *In vivo* doses:20 μg/g	Inhibiting: Bcl-2, Cyclin D1, c-Myc Enhancing: Bax	Inducing cancer cell apoptosis, enhancing immune responses	[567]
**Polysaccharides of *Ulva prolifera***	Non-small cell lung cancer	In *vitro:* A549, *In vitro* doses:400 μg/ml	Inhibiting: MMP-9, p-JNK-2, p-JNK-1, p-ERK-1, p-ERK-2, p-p38, c-Myc	Suppressing cancer cell proliferation and invasion	[562]
**Resveratrol**	Breast cancer, Gastric cancer	*In vitro*: MDA-MB-231, SGC7901, BCap37; *In vitro* doses: 6.25-200 μM, *In vivo*: SGC7901 tumor-bearing mice, BCap37 tumor-bearing mice; *In vivo* doses: 25-100 mg/kg	Inhibiting: c-MYC, cyclin D1, cyclin B1, MMP-2, MMP-9, p-Akt, Sox2, Bmi-1, CD44, p21, p-mTOR, p-Akt, β-cantenin, Wnt 3a, Fibronectin, ZO-1, α-SMA, miRNA-17 Enhancing: Bax, MLKL, p62, VDAC1, LC3, Beclin 1, ATG3, ATG5, p-p38, p-ERK, CHOP, BAP31	Suppressing tumor stem cell functions, inducing apoptosis, inhibiting tumor cell proliferation and invasion, promoting adaptive immunity	[584-586]
**Shikonin**	Leukemia, Breast cancer	*In vitro*: U937, NB4, Namalwa, Raji, MCF-7, T47D, MDA-MB-231; *In vitro* doses: 0.1-10 μM *In vivo*: Namalwa tumor-bearing mice; *In vivo* doses: 4mg/kg	Inhibiting: c-Myc, p-Akt, p-ERK1/2, YAP1, Glut1, Bcl-2, miRNA-19a, PI3K, p-mTOR, p70, ERα Enhancing: p-SAPK/JNK, cleaved caspase-3, cleaved caspase-9, cleaved PARP, p-P38, p-JNK	Inducing cancer cell apoptosis, arresting cell cycle, synergizing with anti-cancer drugs	[571-574]
**Triptolide**	Colorectal cancer, Head and neck cancer, Osteosarcoma, T-cell acute lymphoblastic leukaemia, Pancreatic cancer	*In vitro*: HCT116, HT29, HK1, FaDu, C666-1, KB, IM-9, MES/SA, MG-63, Jurkat, Molt4; *In vitro* doses: 5-200 Nm; *In vivo*: HK1 tumor-bearing mice; P4057 tumor-bearing mice; *In vivo* doses: 0.1 mg/kg,	Inhibiting: cyclin A, cyclin C, cyclin D1, cyclin D3, N-Myc, c-Myc, COX-2, TIE2, VEGF, NRF2, SLC7A11, RPB1, P-gp, SOX2, HIF-1α, β-catenin, TCF7, DNMT1, DNMT3a Enhancing: initiation of GSDME, Bax, Bad, Bak1, cleaved caspase-3, cleaved PARP, ratio of LC3 II/I, Beclin1	Inhibiting tumor cell invasion, inducing pyroptosis, reversing multiple drug resistance	[575-578]

## 5. Remarks and Further Perspectives

Numerous recent studies have investigated MYC oncogenicity, particularly in the field of tumorigenesis. Even temporary MYC inhibition appears to halt tumor induction and ectopic proliferation, suggesting that strategies for MYC inhibition have theranostic potential for tackling MYC-driven cancers; however, direct MYC inhibition is challenging, as the protein lacks an active binding site for small molecules. Alternatively, the development of techniques to inhibit MYC intermediates (e.g., BET, MCL-1, BCR, and CDK) are recommended as a viable approach to indirectly suppress MYC. Thus, identifications of these intermediate pathways are necessary to explore the establishment of more specific and less toxic agents for application in cancer therapy. In this article, we discuss several promising patents/strategies for inhibiting MYC. Additionally, complete eradication of the biological functions mediated by MYC would be challenging, since they are vital to cell function. For example, MDR frequently occurs in carcinomas overexpressing MYC, in which MYC increases the expression levels of efflux transporter proteins, promotes drug-repressor proteins, and reduces MYC corepressor proteins. These chain reactions cause drug delay, non-specific delivery, or drug-specific incapacity. Hence, MYC-induced MDR can arise due to aberrant gene expression at several points. Thus, combination treatment is highly recommended, to modulate MYC levels through alternative protein expression levels/complexes, with the aim of overcoming MDR induction.

An important obstacle to the long-term efficacy of MYC inhibitors is the development and spread of drug resistance. Treatment development mostly involves identification of a drug or effector that blocks one or two MYC-related pathways associated with tumorigenesis, but long-term selective pressure caused by blocking a specific MYC pathway may lead to emergence of drug resistance, allowing cancerous cells to bypass the targeted pathways and proliferate immortally in an alternative way. Many studies have presented evidence that patients only have a better short-term prognosis, while most experience development of refractory disease or cancer recurrence, progressing to MDR. Innovations involving augmented co-factors, combining antitumor agents and super inhibitors, such as THZ1 and JQ1 (direct MYC inhibitors) with self-immune response, to modulate multiple signaling pathways simultaneously, may prevent cancer recurrence and progression to MDR in the long-term [587]. In such co-treatments, MYC-driven cells can undergo apoptosis, autophagy, pyroptosis, or ferroptosis, ensuring cancer cell elimination, even if they escape one of the targeted pathways. Hence, the efficacy of direct MYC targeting and drug-combination co-treatment should be evaluated in future clinical studies, to determine whether it could be a potent therapeutic tool to overcome MYC-induced MDR or off-target effects.

Research into personalized medicine approaches, such as herbal medicines and marine drugs, which are associated with better prognosis (less drug toxicity and higher bioavailability), is increasing for patients with various types of cancer. In recent years, marine drugs have also emerged as a promising source of novel compounds with therapeutic potential. For instance, deoxynyboquinone and its derivative, isobutyl-deoxynyboquinone, have demonstrated significant biological activity, particularly in the field of cancer treatment. Both compounds can inhibit the growth of cancer cells and induce apoptosis, making marine drugs a potential candidate for the development of new cancer drugs. Hence, MYC may be an attractive candidate for effectively controlling tumorigenesis by examination of either protein-protein or protein-small molecule (e.g., ions, MADs, MIZ1, and miRNAs) interactions, to manipulate the balance of MYC expression in cancer cells. Certain marine drugs and herb medicines have the potential to modulate MYC activity and inhibit tumor growth.

In this review, we discuss several articles reporting how herbal medicines can function as modulators of immune checkpoints, mediating MYC-associated cancer oncogenes to optimize immunometabolism, with fewer side effects and better prognosis. Hence, co-treatment of patients with MYC-induced cancer using conventional therapeutic approaches in conjunction with herbal medicine may become a future trend in cancer therapeutics. Several herbal medicines have progressed to clinical trials. Specifically, berberine and curcumin are in phase I and II trials for treating ulcerative colitis and colorectal neoplasia, respectively [588, 589]. Furthermore, National Medical Products Administration of China has approved icaritin for use in the treatment of hepatocellular carcinoma and ginsenoside RG3 has passed phase II clinical trials and is undergoing phase III clinical investigation for treatment of primary liver cancer [590]; however, the number of studies investigating the pharmacodynamics of herbal medicine remains limitedl. Further study of the roles of herbal medicine in influencing MYC, and quantitative data on their toxicity and bioavailability from human clinical trials, are urgently needed.

## 6. Conclusions

In this review, we comprehensively summarize the multiple biological functions of the MYC oncoprotein in cancer treatment and discuss the multifunctional capacity of MYC in various cellular cancer processes, including its influences on immune response, metabolism, cell cycle, apoptosis, autophagy, pyroptosis, metastasis, angiogenesis, multidrug resistance, and intestinal flora, among others. Finally, we describe pending challenges and future perspectives in biomedical research involving the development of therapeutic approaches to modulate MYC or its targets. Overall, further breakthrough investigations are needed, which may provide new insights into MYC functions in tumorigenesis and lead to development of novel therapeutic agents/inhibitors that specifically target MYC-driven tumors.
